# A salt-driven mechanism for precise chirality sorting of carbon nanotubes

**DOI:** 10.1126/sciadv.adx3958

**Published:** 2025-07-11

**Authors:** Min Lyu, Cheng Li, Yanzhao Liu, Yan Li, Ming Zheng

**Affiliations:** ^1^ Beijing National Laboratory for Molecular Science, Key Laboratory for the Physics and Chemistry of Nanodevices, College of Chemistry and Molecular Engineering, Peking University, Beijing 100871, China.; ^2^ Materials Science and Engineering Division, National Institute of Standards and Technology, 100 Bureau Drive, Gaithersburg, MD 20899, United States.

## Abstract

Sorting single-wall carbon nanotubes (SWCNTs) by chirality/handedness is of substantial scientific and technological importance. Existing sorting methods often lack simplicity, reproducibility and scalability. Here, we demonstrate a salt-driven SWCNT sorting mechanism that addresses these challenges. Various polyethylene glycol (PEG)/salt aqueous two-phase systems effectively modulate the top-phase partitioning of DNA-wrapped SWCNTs (DNA-SWCNTs) across a broad range of hydrophobicities, with cation composition markedly affecting top-phase partitioning in the order NH_4_^+^ > K^+^ > Li^+^ > Na^+^ closely resembles the well-documented Hofmeister series. We designed PEG/salt systems with defined [K^+^]:[Na^+^] cation ratios to precisely regulate SWCNT partitioning, enabling one-step top-extraction of multiple single-chirality SWCNTs. The cation ratio parameter, determined in small-scale experiments, remains effective at scales 200 times larger, allowing one-step, milligram-scale separation of (−) (6,5) with record-high enantiomeric purity. In addition, we develop a salt-switching multistage bottom-extraction strategy to isolate (+) (6,5) enantiomer. Our method uses simple salts to provide an easy-to-implement, high-precision, scale-invariant, and cost-effective platform for routine SWCNT chirality sorting.

## Teaser

A salt-driven method enables efficient, precise, cost-effective, and scalable sorting of SWCNTs by chirality and handedness.

## INTRODUCTION

The structure (i.e., chirality) of a single-wall carbon nanotube (SWCNT), defined by the roll-up vector on two-dimensional graphene lattice and denoted by (*n*, *m*), determines all its intrinsic physical properties. Single-chirality SWCNTs have shown great potential in various applications, including high-performance electronic devices (*1*–*5*), single-photon quantum emitters (*6*–*10*), sensing (*11*–*16*), and imaging (*17*–*20*). However, the structural diversity existing in commercially available SWCNTs and those synthesized in laboratories makes chirality sorting an indispensable prerequisite for many fundamental studies and applications (*21*, *22*). Chirality sorting is pivotal in circumventing the performance inconsistencies and property discrepancies that inevitably emerge from the presence of mixed chiral structures, thereby ensuring the capability and reliability of SWCNT-based technologies.

Over the past two decades, several methods have been developed for SWCNT separation, including ion exchange chromatography (*23*–*25*), density gradient ultracentrifugation (*26*–*29*), selective extraction by conjugated polymers (*30*–*34*), gel chromatography (*35*–*39*), and aqueous two-phase extraction (ATPE) (*40*–*47*). Among these, ATPE has emerged as a highly efficient approach due to its ability to separate SWCNTs based on differential partitioning in top and bottom phases, driven by minimal hydrophobicity and hydrophilicity differences. This method has demonstrated substantial scalability and has been successfully applied to the sorting of both surfactant-dispersed and DNA-dispersed SWCNTs (DNA-SWCNTs). Because of the vast expanse of the single-stranded DNA (ssDNA; hereinafter referred to as DNA) library, in company with the sequence-specific interaction between DNA and structurally diverse SWCNTs, DNA and SWCNTs form an intriguing family of hybrid molecular structures, which enables not only the efficient ATPE of single-chirality SWCNTs but also applications in DNA-directed programmable assembly of SWCNTs toward complex devices and circuits (*48*–*51*) and molecular sensing to achieve single-molecule sensitivity and multiplex detection capability (*11*, *12*, *15*, *52*, *53*). Despite these advantages, ATPE sorting of DNA-SWCNTs has predominantly relied on polymer/polymer systems, which face several limitations: (i) The need to introduce modulating agents, such as polyvinylpyrrolidone (PVP), complicates the sorting process and can compromise resolution due to potential interactions with DNA-SWCNT hybrids. (ii) Variability in the chemical purity of polymer phase components—caused by polydispersity, batch-to-batch variations, and trace impurities—affects the consistency and reliability of the sorting process, which is particularly evident for the bottom phase–forming polymers such as polyacrylamide (*43*, *45*) and dextran (*42*) in the aqueous two-phase (ATP) system we have developed in the past. These challenges create notable barriers to the widespread adoption of ATPE as a routine laboratory procedure for single-chirality SWCNT preparation.

In addition to polymer/polymer ATP systems, polymer/salt ATP systems, especially polyethylene glycol (PEG)/salt systems, first reported by Albertsson in 1956 (*54*), have also been extensively studied for the separation of substances from biomolecules to metal ions to nanomaterials (*55*–*58*). They hold promise for large-scale/industrial applications (*56*, *59*–*61*) due to the replacement of the bottom phase–forming polymer with cost-effective low–molecular weight salt, offering advantages such as lower viscosity and shorter phase separation time and lower cost.

Here, we report an expected simple replacement of polymer-polymer two-phase systems with polymer-salt two-phase systems that addresses each of the issues outlined above and enables a salt-driven SWCNT sorting mechanism. We demonstrate this mechanism using PEG/salt ATP systems for the sorting of DNA-SWCNTs. A key finding of our study is the identification of a cation-dependent modulation of SWCNT partitioning, analogous to the well-documented Hofmeister phenomenon. Leveraging this finding, we have developed methods for single-step extraction of multiple enantiomerically pure nanotube structures and a multistage protocol for the large-scale separation of SWCNTs. This approach substantially simplifies the ATP separation process for DNA-SWCNTs, improving its reproducibility, scalability, and cost-effectiveness. We believe that our findings will accelerate the widespread adoption and implementation of SWCNT sorting techniques, fostering advancements in fundamental research and applications.

## RESULTS

### Salt-dependent spontaneous partition of SWCNTs in PEG/salt ATP systems

**Fig. 1.**
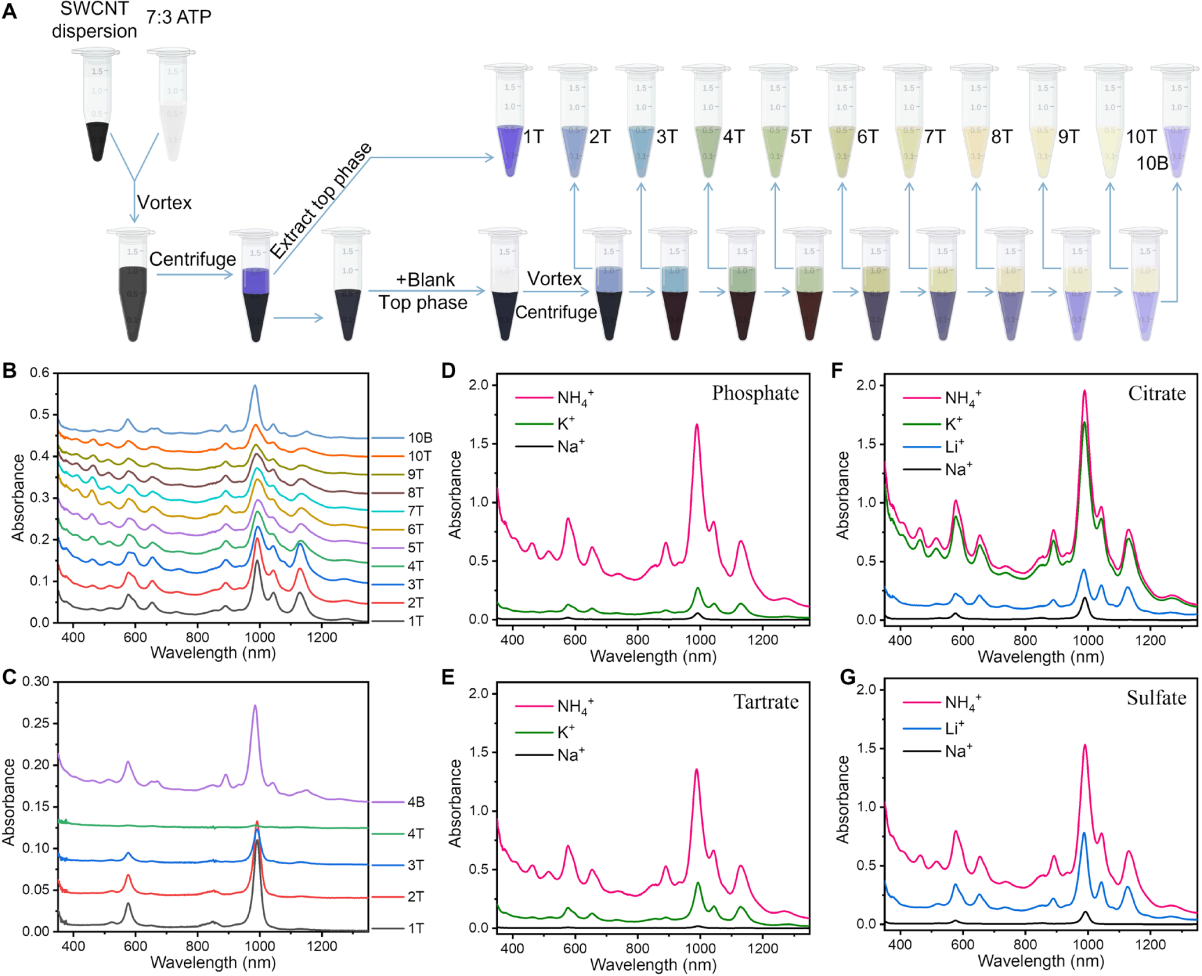
Cation-dependent partitioning of 65ss-SWCNTs in PEG/salt ATP systems. (**A**) Scheme showing the ATPE steps of 65ss-SWCNTs in PEG/potassium phosphate ATP system (interfacial trapping is not shown). Absorption spectra of each fraction obtained during the sorting of 65ss-SWCNTs in (**B**) PEG/potassium phosphate ATP system and (**C**) PEG/sodium phosphate ATP system. Absorbance spectra of the first top fraction spontaneously partition to the top phase after loading 65ss-SWCNTs into (**D**) PEG/ammonium phosphate, PEG/potassium phosphate, and PEG/sodium phosphate ATP systems; (**E**) PEG/ammonium tartrate, PEG/potassium tartrate, and PEG/sodium tartrate ATP systems; (**F**) PEG/ammonium citrate, PEG/potassium citrate, PEG/lithium citrate, and PEG/sodium citrate ATP systems; and (**G**) PEG/ammonium sulfate, PEG/lithium sulfate, and PEG/sodium sulfate ATP systems.

Many kinds of salt can form aqueous two-phase systems with PEG in which the top phase is enriched with PEG and the bottom phase is enriched with salt. As shown in Table 1, we have studied the sorting of DNA-SWCNTs in 13 kinds of PEG/salt ATP systems. Preparation details of the PEG/salt ATP system are listed in Materials and Methods. The meaning of ATP system (10:0) and ATP system (7:3) and our method for determining their composition based on the phase diagram characteristics of ATP systems are described in detail in fig. S1. A representative example of the critical differences in selectivity driven by the cation choice for the salt used is shown in Fig. 1 (B and C) using sodium phosphate and potassium phosphate.

**Table 1.** Composition of ATP systems (10:0) used in this work.

ATP system		Cation or anion	Concentration of phase-forming salt (mol∙liter^−1^)	[PEG]/g∙liter^−1^
		[Li^+^], [Na^+^], [K^+^], or [NH_4_^+^]	∑[salt anions]*	
PEG6k/phosphate†	Na	0.945	0.600	94.920
	K	1.05	0.666	105.420
	NH_4_	1.33	0.664	104.160
PEG6k/citrate	Na	1.26	0.419	115.920
	Li	2.17	0.722	130.560
	K	1.44	0.482	105.000
	NH_4_	1.83	0.609	119.700
PEG6k/tartrate‡	Na	1.19	0.594	113.820
	K	1.36	0.678	119.700
	NH_4_	1.79	0.895	123.060
PEG6k/sulfate§	Na	1.09	0.547	100.380
	Li	1.69	0.847	126.000
	NH_4_	1.60	0.805	103.320

*The anion concentration represents the sum of the concentrations of all ionized forms of phosphate, citrate, or tartrate ions in solution.

†PEG/lithium phosphate (7:3) cannot be prepared due to the poor solubility of lithium phosphate.

‡PEG/lithium tartrate (7:3) cannot be prepared due to the poor solubility of lithium tartrate.

§PEG/potassium sulfate (7:3) cannot be prepared due to the poor solubility of potassium sulfate.

The primary parent samples used in this work are two highly stable SWCNT dispersions (65ss-SG65i and 83ss-SG65i, hereinafter referred to as 65ss-SWCNTs and 83ss-SWCNTs) prepared with previously identified DNA sequences as the SWCNT dispersant, which enabled the exceptional separation of left- and right-handed (6,5) and (8,3) enantiomers, respectively, using polymer/polymer ATP systems (*43*)—A/T-based (65ss, TTATATTATATT) and C/T-based (83ss, TTTCCCTTTCCCCCC). Typical absorbance spectrum and photoluminescence two-dimensional (2D) excitation-emission map of the initial DNA-SWCNT dispersion are shown in fig. S2. We first study the partition of 65ss-SWCNTs and 83ss-SWCNTs in the PEG/potassium phosphate (pH = 7) ATP system. Similar to polymer-polymer systems, PEG-salt mixtures can exhibit phase transition into a two-phase system above critical polymer and salts concentrations. Figure 1A illustrates selective fractionation by the ATPE process using the 65ss-SWCNT dispersion as an example. By mixing the dispersion with the 7:3 ATP stock solution and then centrifugating to expedite phase separation, (6,5)-enriched SWCNTs spontaneously partition to the top phase (1T, absorbance spectrum shown in Fig. 1B), while some DNA-SWCNTs are trapped at the interface. After the extraction of 1T, the remaining bottom phase is mixed with an equal volume of blank top phase and then DNA-SWCNTs with lower (6,5) enrichment distribute to the top phase after phase separation. After repetitive extraction of top phase and the addition of blank top phase, DNA-SWCNTs continue to distribute to the top phase, while increasing amounts of SWCNTs are trapped by the interface. Last, what remains in the bottom are also (6,5)-enriched SWCNTs. For the sorting of 65ss-SWCNTs in the PEG/sodium phosphate (pH = 7) ATP system, single-chirality (6,5) SWCNTs spontaneously partition to the top phase (1T, absorbance spectrum shown in Fig. 1C), while most SWCNTs remain in the bottom phase. After another two steps of extraction of top phase and the addition of blank top phase, highly enriched (6,5) continues to distribute to the top phase, while most SWCNTs still remain in the bottom phase with no tendency of partitioning to the top phase. With the addition of 0.5 μl of 20% (mass/mass) PVP40k in water, most SWCNTs are trapped by the interface, and what remains in the bottom are (6,5)-enriched SWCNTs. The sorting of 83ss-SWCNTs in the two PEG/salt systems above is shown in fig. S3.

The strikingly distinct partitioning behavior of DNA-SWCNTs observed between PEG/potassium phosphate and PEG/sodium phosphate ATP systems strongly suggests that the identity of the salt or cation plays a substantial role in controlling DNA-SWCNT distribution within PEG/salt systems, which was also reported in polymer/polymer ATP systems (*42*, *45*, *47*, *62*–*64*). Based on the 65ss-SWCNT dispersion, we have studied and compared its partition pattern across 13 kinds of PEG/Salt ATP systems systematically by measuring the absorbance spectra of those SWCNTs that spontaneously partition to the top phase. The results demonstrate that the distribution of DNA-SWCNTs in PEG/salt ATP systems is strongly modulated by the phase-forming salts.

We divide the partition results into four groups according to the type of anions as shown in the Fig. 1 (D to G). Take the comparison in PEG/phosphate system (Fig. 1D) as an example, according to the concentration of DNA-SWCNTs spontaneously distributed to the top phase (1T), as measured by ultraviolet–visible–near infrared (UV-Vis-NIR) absorbance, PEG/ammonium phosphate is much stronger than the other salts shown at driving partitioning to the PEG phase, with PEG/potassium phosphate being greater than PEG/sodium phosphate among the remaining two. The other three groups exhibit similar trends. Considering the results across four groups, the modulation capability of cations, which refers to their effect on the amount of DNA-SWCNTs distributed to the top phase, is arranged as NH_4_^+^ > K^+^ > Li^+^ > Na^+^.

In a similar way, the partition results can be divided into another four groups according to the type of cations as shown in the fig. S4 (A to D). However, the modulation capability of anions is not as consistent as that of cations across the four groups. For instance, in PEG/sodium salt group compared to the PEG/potassium group, the order of phosphate and tartrate is reversed. Similar inversions in strength of effect were also observed in other comparisons examining different anions, implying that the effects of anion selection are more complex than cation effects.

### One-step extraction of single-chirality (6,5) and (8,3) enantiomers

A careful check of Fig. 1 shows the selective sorting of (6,5) tubes in all four sodium salt systems. However, the concentrations are very low. To realize an efficient chirality dependent sorting, we needed to further optimize the sorting condition. Because K^+^ provide slightly stronger modulation capability than Na^+^, using salt with a tuned mixture of Na^+^ and K^+^ cations would increase yield while retaining high selectivity and simultaneously expanding the operational parameter window for precise control. Rochelle salt (potassium sodium tartrate) offers a convenient option for making an ATP system with an equal amount of K^+^ and Na^+^. Although the enrichment of (6,5) isolated from the top phase was very high, obvious transition peaks contributed by (7,5) as contaminant could still be distinguished in the absorption spectrum reported in fig. S5. In addition, these commercially available double cation salts are fairly rare for other salt anions, and there is not room left for optimizing the composition of cations in such a system. Thus, the cation composition in different PEG/salt ATP systems should be further optimized. For the convenience of subsequent discussion, here we define a dimensionless parameter termed “K/Na ratio,” which equals to [K^+^]:[Na^+^] for a given PEG/salt system.

We focus on optimizing the K/Na ratio in PEG/phosphate ATP systems driven by our observation that DNA-SWCNTs in a phosphate medium demonstrate higher stability than other salt mediums and the appropriate modulation ability of K^+^ and Na^+^. First, aqueous solutions of potassium phosphate buffer (KPB, pH = 7, [PO_4_^3−^] + [HPO_4_^2−^] + [H_2_PO_4_^−^] = 3 mol/liter) and sodium phosphate buffer (NaPB, pH = 7, [PO_4_^3−^] + [HPO_4_^2−^] + [H_2_PO_4_^−^] = 3 mol/liter) are prepared. Phosphate buffer solution with a specific cation ratio was prepared by mixing the KPB and NaPB in a corresponding volume ratio. Phase separation conditions for each K/Na ratio are explored based on the previously published procedures (*42*), with ATP solutions prepared according to the recipe provided in Table 1 and table S1.

**Fig. 2.**
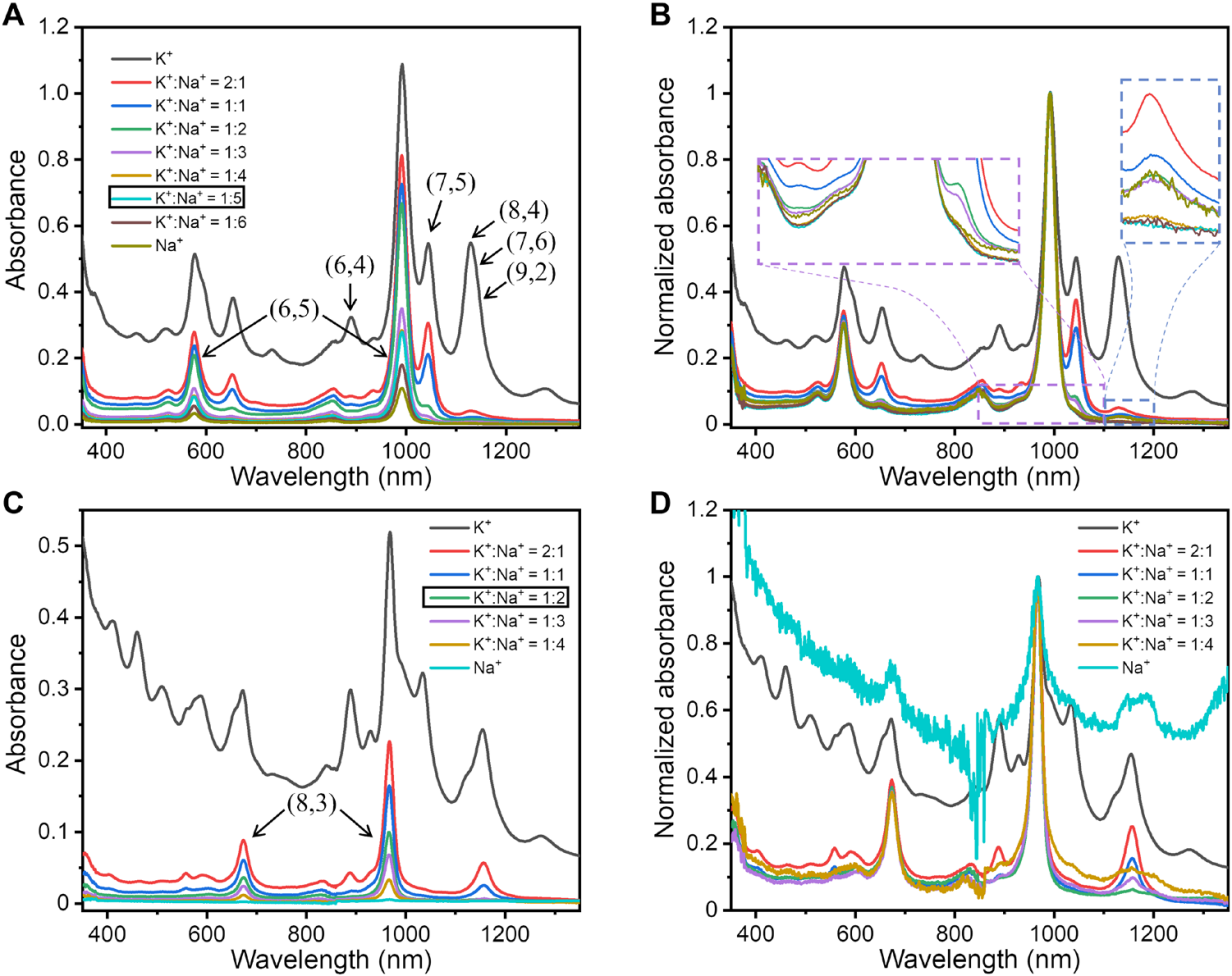
Optimization of the best K/Na ratio for the single-step extraction of DNA-SWCNTs. (**A**) Absorbance spectra and (**B**) Normalized absorbance spectra of the first top fraction spontaneously partition to the top phase after loading 65ss-SWCNTs into the PEG/phosphate ATP system with different K/Na ratios. The inset shows the enlarged baseline area of the (6,5) *E*_11_ transition peak. The best condition for extracting (6,5) is marked with a black square. (**C**) Absorbance spectra and (**D**) normalized absorbance spectra of the first top fraction spontaneously partition to the top phase after loading 83ss-SWCNTs into the PEG/phosphate ATP system with different K/Na ratios. The best condition for extracting (8,3) is marked with a black square.

**Fig. 3.**
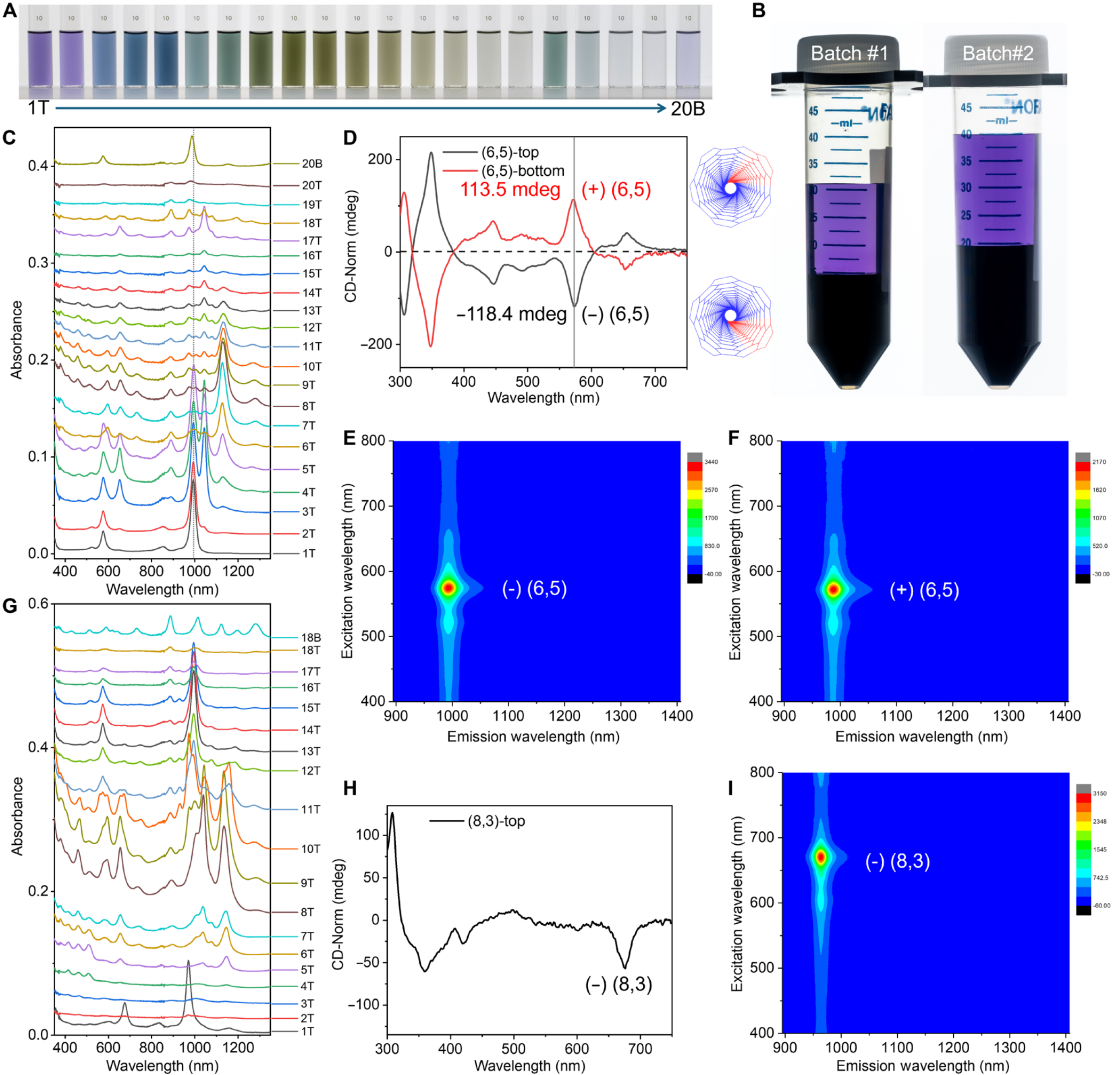
Multistage sorting enabled by salt-switching extraction. (**A**) Optical photographs of each fraction obtained during the ≈30-ml scale sorting of 65ss-SWCNTs. Fraction numbers, from left to right, are 1T, 2T, ... to 20B. The “10” above each fraction’s liquid level is a 10 ml mark on the colorimetric tube. (**B**) Photographs of the ≈30-ml scale (Batch #1) and ≈40-ml scale (Batch #2) aqueous two-phase system after the first step of centrifugation and phase separation. The purple transparent part in the left centrifuge tube is the top phase 1T in (C). (**C**) Absorption spectra of each fraction obtained throughout the ≈30-ml scale sorting of 65ss-SWCNTs commenced with the PEG/phosphate ([K^+^]: [Na^+^] = 1:5) ATP system, followed by using the top phase of PEG/potassium phosphate as fresh top phase from the 2nd to the 16th step and the top phase of PEG/potassium citrate as fresh top phase from the 17th to the last step. (**D**) Normalized CD spectra (normalized by the *E*_22_ absorbance of the corresponding fraction) of (6,5) enantiomers isolated from 1T fraction and 20B fraction of the multistage sorting shown in (A). Photoluminescence 2D excitation-emission maps of the (**E**) (−) (6,5) and (**F**) (+) (6,5) enantiomers (referenced by the sign of their *E*_22_ CD peaks; solid gray vertical line). (**G**) Absorption spectra of each fraction obtained throughout the sorting of 83ss-SWCNTs commenced with the PEG/phosphate ([K^+^]: [Na^+^] = 1:2) ATP system followed by using the top phase of PEG/potassium phosphate as fresh top phase from the second to the seventh step and the top phase of PEG/ammonium phosphate dibasic as fresh top phase from the eighth to the last step. (**H**) Normalized CD spectra (normalized by the *E*_22_ absorbance of the corresponding fraction) of (−) (8,3) enantiomer isolated from 1T fraction of (G). (**I**) Photoluminescence 2D excitation-emission maps of the (−) (8,3).

For 65ss-SWCNTs, in a series of PEG/phosphate ATP systems with different K/Na ratios, there is an obvious trend of decreasing SWCNT concentration fractionated to the top phase (1T) with the increasing ratio of Na^+^ in Fig. 2A. When KPB is the exclusive component of the phase-forming phosphate salt, (6,5) along with many other species of different chiralities spontaneously distributed to the top phase (absorption spectrum shown as the black curve in Fig. 2A). With the decrease of the K/Na ratio, SWCNT species other than (6,5) distributed in the top phase [such as (6,4), (7,6), (8,4), and (9,2)] markedly decrease, which means that the relative enrichment of (6,5) was increased. As shown by the original absorption spectra in Fig. 2A and the normalized spectra in Fig. 2B, SWCNTs are extracted with the highest (6,5) enrichment/purity and concentration at a K/Na ratio of 1:5. At this condition, for the method and concentration of 65ss-SWCNTs used in this work, an apex value of ~5.56 optical density (OD) (peak absorbance at 991 nm, in cuvette with 1-cm optical path length) single chirality (6,5) can be separated from the top phase with one-step extraction. Further decease in the K/Na ratio contributes negligibly to enhancing the enrichment/purity of (6,5) but leads to substantial reductions in the concentration of (6,5). This indicates that the 1:5 ratio is near the optimal condition. When NaPB is the exclusive component of the phase-forming salt, only a small amount of (6,5) appears in the top phase. Notably, we find that the K/Na ratio determined by the small-scale separation experiment remains valid for larger-scale separation. To demonstrate this, two batches of one-step large-scale extraction in the PEG/phosphate ATP system with a K/Na ratio of 1:5 (Batch #1, ≈30-ml scale sorting shown in Fig. 3, B and C; Batch #2, ≈ 40-ml scale sorting shown in Fig. 3B and fig. S10A) were implemented, yielding ~1.1 mg of single chirality (6,5) combined. Notably, these top-phase fractions can be collected, precipitated, and redispersed in 30 mM NaCl to reduce the polymer content and exchange the salt medium according to the previously published method (*45*, *65*), which we demonstrate via the precipitation exchange of the combined 1T sorted batches into a new 10-ml solution of 30 mM NaCl (fig. S10B). Using this concentrated dispersion, the (6,5) species separated from the 1T top phase were determined to be composed of near entirely the (−) (6,5) enantiomer, with, moreover, the highest enantiomeric purity reported to date [as determined from the circular dichroism (CD) spectrum shown as the black curve in Fig. 3D)] to be further discussed in the next section.

Similar trends are also observed for application of PEG/salt ATPE to 83ss-SWCNTs (Fig. 2C). As shown by the original absorption spectra in Fig. 2C and the normalized spectra in Fig. 2D, when the K/Na ratio is set to 1:2, the purity and concentration of (8,3) SWCNTs in the top phase reach their greatest observed value. Under this condition, for the method and concentration of 83ss-SWCNTs used in this work, an apex value of ~2.00 OD (peak absorbance at 966 nm, in cuvette with 1-cm optical path length) single-chirality (8,3) can be separated from the top phase with one-step extraction. Further decreasing the K/Na ratio in this case is detrimental to the enrichment/purity and for the concentration of (8,3) SWCNTs distributing into the top phase. When NaPB is the exclusive component of the phase-forming phosphate salt, no SWCNTs appears in the top phase. The (8,3) separated from top phase is (−) (8,3) enriched (CD spectrum shown in Fig. 3H). Similar trends were also observed for the separation of (9,1) using CTTC_3_TTC-SWCNT dispersion reported in fig. S6. The best separation condition of (9,1) used NaPB as the exclusive component of the phase-forming salt.

These findings demonstrate that the precise modulation of the K/Na ratio not only is a critical step in optimizing the one-step separation process but also likely represents a universal strategy for achieving the optimal separation conditions toward specific (*n*, *m*) species. The universality of this approach is evident, as it is applicable across various resolving DNA sequences (i.e., sequences that form a specific wrapping structure on specific SWCNT species to enable their separation) and different salt systems, such as PEG/tartrate and PEG/citrate systems (figs. S7 and S8).

### Multistage separation enabled by salt switching

A second manner of separating SWCNT species is to conduct multiple consecutive ATPE steps, recovering additional SWCNT species from the intermediate fractions or the final bottom fraction. Typical multistage separation involves a repetitive sequence of steps: supplementing the fresh top phase, introducing a modulating agent to control the distribution of SWCNTs, mixing the solution by vortex to ensure sufficient mass transfer, centrifuging to drive complete phase separation, and subsequently extracting the top phase. The top phase for the nth extraction cycle is denoted as nT. Multistage extraction of 65ss-SWCNTs in the PEG/KPB ATP system, shown in Fig. 1A, leaves a (6,5)-enriched bottom fraction (10B), which is hard to further purify to obtain single-chirality (6,5).

Through the study of anion-dependent partition (fig. S4), we observed that the tendency of DNA-SWCNTs to spontaneously partition to the top phase in the PEG/potassium citrate ATP system is much greater than that in the PEG/KPB ATP system. Therefore, we hypothesized that replacing a blank top phase of the PEG/KPB system with a blank top phase of the PEG/potassium citrate system during a multistage process would gradually increase the concentration of citrate in the system and would enhance the ability of the DNA-SWCNTs to partition to the top phase. Because no traditional modulating agent such as PVP is used in this sorting route, the structural selectivity of the separation during the later sorting steps should be improved.

For 65ss-SWCNTs, after the extraction of 1T as shown in Fig. 3C, a blank top phase of PEG/KPB was used to mix with the remaining bottom phase, driving more SWCNTs to partition to the top phase. As the sorting proceeded to the 16th step, the trend of additional SWCNTs partition to the top phase decreased markedly. After the extraction of 16T, a blank top phase of PEG/potassium citrate was used to resume the trend of SWCNTs to partition to the top phase. After another four-step extraction, SWCNTs partition to the top phase demonstrate progressive enrichment of (6,5), while those finally retained in the bottom phase (20B) achieve a single-chirality (6,5) comparable to the (6,5) obtained in 1T. Photographs and absorption spectra of each fraction obtained during the sorting process are shown in Fig. 3 (A and C). Notably, the absorption peak corresponding to the *E*_11_ optical transition of (6,5) shows a 6-nm shift between the 1T (6,5) and the 20B (6,5) fractions.

CD spectra confirmed that these fractions are dominatingly composed of opposing enantiomers, i.e., of opposite carbon lattice twist direction. Figure 3D displays the CD spectra of the single-chirality (6,5) sorted from 1T and 20B, normalized by their absorption intensities at the *E*_22_ transition. The normalized CD intensities of both (6,5) enantiomers, −118.4 and 113.5 mdeg, have both exceeded the highest values reported in the literature (*35*) to the best of our knowledge, indicating that the two (6,5) enantiomers obtained by this sorting method have the highest enantiomeric purity known so far. The two (6,5) enantiomers with the highest enantiomeric purity to date enabled by one-step extraction fully demonstrate the superiority of the separation method based on ATPE of DNA-SWCNTs in PEG/salt systems. Their photoluminescence excitation-emission spectra for the same fractions are shown in Fig. 3 (E and F). With another batch of ≈40-ml scale separation (Batch #2; Fig. 3B and fig. S10A), ≈1.1 mg (−) (6,5) and ≈0.23 mg (+) (6,5) are separated [using the published value of 5 μg/ml for OD = 1 at the *E*_11_ transition peak of (6,5) (*45*, *66*)]. It is worth noting that previous method for the separation of high-purity (6,5) enantiomers relies on the combination of multiple separation methods such as length fractionation and gel chromatography. In the figs. S11 to S15, we provide an analysis showing that a PEG/salt system has a higher ability to enhance longer nanotube extraction than a polymer/polymer system due to much more uniform mass distribution of salt. We propose that this attribute is responsible for the higher enantiomeric purity we have achieved because longer tubes are observed to have less absorbance between interband optical transitions (baseline absorbance) for the same peak absorbance in their spectra (*27*, *67*).

This strategy is also applicable for the sorting of 83ss-SWCNTs (Fig. 3G). Starting from the PEG/phosphate system with a K/Na ratio of 1:2, single-chirality (8,3) is obtained in 1T. Then, the blank top phase of PEG/KPB is used to mix with the remaining bottom phase until the extraction of 7T. Hereafter, a blank top phase of PEG/ammonium phosphate dibasic is used to allow the extraction of the remaining fractions. The absorption spectra of each fraction obtained during the sorting process are shown in Fig. 3G. The CD spectrum and photoluminescence 2D excitation-emission map of the single-chirality (8,3) sorted from 1T are shown in Fig. 3 (H and I, respectively). Although, another kind of (8,3) enantiomers cannot be separated from the bottom, the later fractions such as 13T and 14T demonstrate some selectivity toward (6,5).

Because the SWCNTs from the final bottom phase are obtained through multistage sorting, the separation outcome is considerably affected by the extraction procedure and the stability of DNA-SWCNT dispersion. The multistage extraction of the 65ss-SWCNTs reliably results in the separation of single-chirality (6,5) from the final bottom phase, with the opposite handedness of (6,5) in 1T. However, the isolation of (8,3) SWCNTs in the bottom phase from the less stable 83ss-SWCNTs dispersion has invariably met with failure. This is probably attributed to the lesser abundance of (8,3) than (6,5) in the starting SG65i SWCNT population and the severe interfacial trapping/precipitation caused by the high ionic strength of PEG/salt systems. The latter effect is particularly pronounced for the 83ss-SWCNT dispersion, which exhibits lesser colloidal stability under high-salt conditions compared to 65ss-SWCNT dispersion. During the entire sorting process shown in Fig. 3 (C and G), interfacial trapping led to a loss of about 26% of the SWCNTs for 65ss-SWCNTs and about 55% of the SWCNTs for 83ss-SWCNTs (inferred from the analysis in fig. S16). The separation purity of (8,3) from the top phase can even be affected in salt systems that strongly influence the colloidal stability of DNA-SWCNT dispersion, such as sulfate systems (fig. S17). This likely explains why some previously identified DNA sequences, which enable selective polymer-polymer ATPE selection, fail to work in PEG/salt systems (figs. S18 to S21). Previously, we found that the stability of DNA-SWCNTs is affected under high salt concentrations (*45*, *68*), prompting us to use high concentration salts to precipitate SWCNTs obtained through ATPE. Interfacial trapping was always unavoidable due to the high concentration of salt, which is a phase-forming component in PEG/salt ATP systems. To date, we have observed the highest robustness in separation outcomes within PEG/phosphate salt ATP systems.

### Mechanism of the cation-dependent partition

Understanding the driving force behind this phenomenon could potentially unveil the principles governing molecular behavior in high salt solutions, an ionic interaction-based, strongly correlated condensed phase.

As a starting point toward this goal, we consider all the pair-wise interactions present in the system. Because the order of cations for DNA-SWCNT partition in the top phase is observed to be consistent across different systems while inconsistent for anions, pair-wise interactions involving salt are discussed exclusively in relation to salt cations hereafter. The order of cations in promoting DNA-SWCNTs’ partition in top phase (NH_4_^+^ > K^+^ > Li^+^ > Na^+^) closely resembles the ranking of kosmotropic cations in Hoffmeister series for their ability to salt out proteins (NH_4_^+^ > K^+^ > Na^+^ > Li^+^) (*69*, *70*). The reversed order of Li^+^ may be attributed to the PEG/cation interaction involved in the system. The lone pair of electrons on the etheric oxygen confers an anionic character to PEG, allowing it to interact with cations. This interaction has been confirmed by nuclear magnetic resonance spectroscopy (*71*) and surface force balance (*72*) and observed to be dependent on the ion size and charge density. For monovalent cations, the hydration level of PEG in chloride salt follows the order Li^+^ > Na^+^ > K^+^ (*73*). This order is opposite to the partition trend of DNA-SWCNTs to the top phase based on the model of solvation energy spectrum proposed to explain the partition of DNA-SWCNTs in ATP systems (*42*, *74*). The strong interaction between Li^+^ with PEG may act as a competing factor to its “salting out” ability by modifying the PEG phase to make it more hydrophilic. This may increase the tendency for DNA-SWCNTs to partition toward the top phase, thereby positioning Li^+^ to the left of Na^+^.

This alignment between the order of cations in promoting DNA-SWCNTs’ partition in top phase and Hoffmeister series suggests a shared mechanistic foundation. Notably, while classical Hofmeister effects typically manifest in bulk protein precipitation, in our system, this effect is reflected in the directional phase partitioning of DNA-SWCNTs. Both phenomena share a common thermodynamic driving force: Salt modulates the solvation energy or solubility of DNA-SWCNTs, similar to its role in protein salting-out processes. We hypothesize that cations with higher salting-out power (as per their Hofmeister position) induce less hydrated DNA-SWCNT structures, thereby enhancing their expulsion from the more hydrophilic, salt-rich bottom phase. In other words, DNA-SWCNTs are “salted out” from the bottom salt-rich phase into the top PEG-rich phase.

**Fig. 4.**
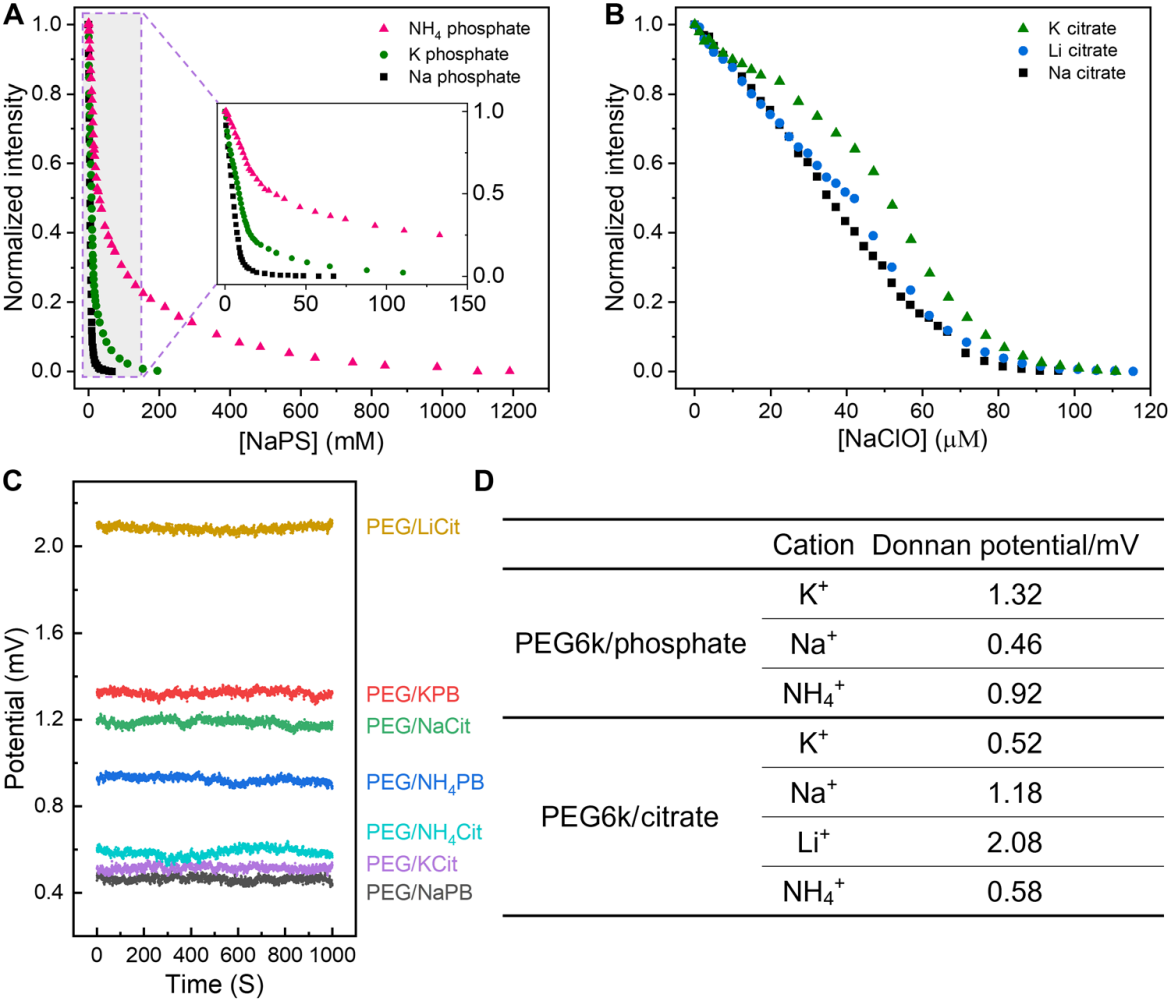
Mechanism of the cation-dependent partition. (**A**) PL intensity of 65ss-SWCNTs (at 988 nm) in sodium phosphate, potassium phosphate, and ammonium phosphate medium quenched by adding NaPS. (**B**) PL intensity of 65ss-SWCNTs (at 988 nm) in sodium citrate, potassium citrate, and lithium citrate medium quenched by NaClO. (**C**) Measured Donnan potential of PEG/potassium phosphate, PEG/sodium phosphate, PEG/ammonium phosphate, PEG/potassium citrate, PEG/sodium citrate, PEG/lithium citrate, and PEG/ammonium citrate ATP systems. (**D**) Summary of the measured Donnan potential of seven ATP systems shown in (C).

The cations’ ability to salt out DNA-SWCNTs can also be inferred indirectly from the ease with which DNA-SWCNTs are oxidized by hydrophilic oxidants such as sodium hypochlorite (NaClO) or sodium persulfate (NaPS). Experimental details can be found in Materials and Methods section and figs. S22 to S27. Figure 4 (A and B) reports the reduction of fluorescence intensity from oxidant addition (quenching profiles) for identical aliquots of 65ss-SWCNTs measured in either phosphate or citrate medium. The difference in photoluminescence (PL) intensity gradually increases with the increase of oxidant concentration. According to these quenching profiles, the order of difficulty in oxidizing (antioxidation) 65ss-SWCNTs in phosphate medium for cations is NH_4_^+^ > K^+^ > Na^+^, while K^+^ > Li^+^ ≳ Na^+^ in citrate medium, the overall order can be arranged as NH_4_^+^ > K^+^ > Li^+^ ≳ Na^+^, which correlates well with the modulation order. One possible explanation regarding the antioxidation order is that the hydration extent of the DNA-SWCNT hybrids governs its antioxidative capacity. Strong kosmotropic cations (e.g., NH_4_^+^) competitively withdraw interfacial water molecules, reducing DNA-SWCNT hydration. This dehydration may promote hydrophobic interaction and compacts the DNA-wrapping structure on SWCNTs, limiting oxidant access to the SWCNT surface and enhancing its resistance against oxidant (consistent with NH_4_^+^'s top ranking in both media). Weaker cations (e.g., Na^+^) retain more hydrated, open structures, facilitating oxidant penetration and lowering antioxidant efficacy. The observed consistent ranking of cations in antioxidation and their modulation order in DNA-SWCNTs’ partition suggests that the “salting-out” capability of cations may be the dominant factor governing the cation-dependent partitioning of DNA-SWCNTs in PEG/salt ATP systems, consistent with the proposed “salting-out” mechanism.

Previous studies have shown that the pH of the system strongly affects the partitioning of both surfactant-dispersed SWCNTs (*75*) and DNA-SWCNTs (*42*) in ATP systems. We measured the pH of all salt solutions (listed in table S3) under the same concentration conditions as used in the sorting experiments. While sodium and potassium salts exhibit similar pH values, their modulation capabilities differ markedly. Therefore, we suggest that pH is not the dominant factor affecting separation in this case. For additional discussion on the effect of pH, refer to the Supplementary Materials.

The ionic nature of salt solutions also prompts us to consider spontaneous arising electrical potential differences (Donnan potential) between the top and bottom phases arising from the differential partition of anions and cations between the two phases. Because of the highly charged nature of DNA-SWCNTs (> 5 e/nm) (*76*), for a 1-mV difference and a 250-nm-long tube, the potential difference is = 1 mV × 250 nm × 5 e/nm = 1250 meV ≫ kT (≈25 meV). Any potential difference should thus have a marked effect on DNA-SWCNT partition. We have carried out Donnan potential measurements for a few PEG/salt ATP systems following the method established by Vis *et al.* (*77*), with the measurement details and the data of the entire measurement process under the corresponding electrode settings described in Materials and Methods and Supplementary Materials, respectively.

The measured Donnan potentials (Fig. 4C) containing a total of seven systems in two sets of groups—PEG/phosphate and PEG/citrate—are summarized in Fig. 4D and figs. S28 to S35. The Donnan potential follows the rank K^+^ > NH_4_^+^ > Na^+^ in PEG/phosphate systems while Li^+^ > Na^+^ > NH_4_^+^ > K^+^ in PEG/citrate systems. The ranking within the two sets of PEG/salt systems is inconsistent and also inconsistent with the modulation rank shown in Fig. 1. However, as proposed by Haynes *et al.* (*78*), the measured potential difference here may not represent the actual interfacial electrostatic potential difference for PEG/salt systems due to the non-negligible and asymmetrical liquid junction potential. Besides, previous reports demonstrate that affinity or electrostatic interaction between ligand and target molecules is usually masked by the high ionic strength environment in PEG/salt ATP systems (*79*, *80*).

## DISCUSSION

In this work, we systematically investigate PEG/salt two-phase systems for sorting DNA-SWCNTs. Spontaneous partition of DNA-SWCNTs is observed in these systems, which is found to be modulated by the salt composition, especially the cations. According to the concentration of DNA-SWCNTs distributed in the top phase, the modulation ability of cations follows the rank NH_4_^+^ > K^+^ > Li^+^ > Na^+^, resembling the Hoffmeister series for salting out proteins. For a one-step extraction under optimized cation composition, single-chirality (6,5), (8,3), and (9,1) are separated from the first top fraction. The K/Na ratio control parameter, established through small-scale sorting experiments, remains valid even at separations scaled up by a factor of 200. This has enabled the one-step milligram-scale separation of the (−) (6,5) enantiomer with the highest enantiomeric purity ever reported. In addition, a salt switching strategy is developed to enable multistage extraction, which allows (+) (6,5) of the highest enantiomeric purity to be separated from the bottom phase.

The precise modulation of the K/Na ratio is essential for optimizing the one-step separation process and likely stands as a universal strategy for achieving the optimal separation conditions toward specific (*n*, *m*) species across various resolving DNA sequences and different salt systems. PEG/salt ATP systems hold promise as a highly accurate, scalable, and economical platform for the chiral/enantiomeric separation of DNA-SWCNTs. However, not all of the resolving DNA sequences identified today can be directly switched to PEG/salt systems. The sorting outcome of some DNA-SWCNTs is different from that in the original polymer/polymer ATP system. Many previously identified DNA sequences enabling selective polymer-polymer ATPE selection that were tested suffer from severe interfacial trapping or precipitation during the sorting process. It is therefore necessary to perform targeted screening of such, or new, DNA sequences for their potential use in PEG/salt ATP systems. With single-stage extraction, many sequences can be tested and potentially yield single-chirality species. We hypothesize that screening for DNA sequences or modified DNAs (XNAs) with neutral backbone may yield better resolving abilities in the early fractions or greater colloidal stability to withstand the environment of high salt concentration; these capabilities would contribute to the efficient, large-scale, high-purity, and multistage separation of DNA-SWCNTs in PEG/salt systems. The development of ATPE method based on PEG/salt ATP systems opens the door to more efficient, simpler, and cheaper separation methods for SWCNTs of ultrahigh chiral and enantiomeric purity. This advancement not only establishes standardized separation protocols for SWCNTs but also facilitates investigations into the unique properties of SWCNT enantiomers and their potential applications.

## MATERIALS AND METHODS

### Materials

Certain equipment, instruments, software, or materials, commercial or noncommercial, are identified in this paper to specify the experimental procedure adequately. This identification is not intended to imply recommendation or endorsement of any product or service by NIST nor is it intended to imply that the materials or equipment identified are necessarily the best available for the purpose. ssDNA was purchased from Integrated DNA Technologies and Sangon Biotech. CoMoCAT SWCNT powder (SG65i grade), sodium chloride (NaCl; BDH Chemicals), PEG [molecular weight (MW) of 6 kDa, Alfa Aesar], PVP (MW of 40 kDa, Sigma-Aldrich), sodium phosphate (Na_3_PO_4_, Sigma-Aldrich), sodium phosphate dibasic (Na_2_HPO_4_, Sigma-Aldrich), sodium phosphate monobasic (NaH_2_PO_4_, Sigma-Aldrich), potassium phosphate (K_3_PO_4_, J&K), potassium phosphate dibasic (K_2_HPO_4_, Sigma-Aldrich), potassium phosphate monobasic (KH_2_PO_4_, Sigma-Aldrich), ammonium phosphate dibasic [(NH_4_)_2_HPO_4_, Beijing TongGuang Fine Chemicals], potassium sodium tartrate tetrahydrate (KNaC_4_H_4_O_6_·4H_2_O, Sigma-Aldrich), sodium tartrate dibasic dihydrate (Na_2_C_4_H_4_O_6_·2H_2_O, Sigma-Aldrich), potassium tartrate dibasic hemihydrate (K_2_C_4_H_4_O_6_·0.5H_2_O, Sigma-Aldrich), ammonium tartrate dibasic [(NH_4_)_2_C_4_H_4_O_6_, Sinopharm], potassium citrate tribasic monohydrate (K_3_C_6_H_5_O_7_·H_2_O, Sigma-Aldrich), sodium citrate tribasic dihydrate (Na_3_C_6_H_5_O_7_·2H_2_O, Sigma-Aldrich), ammonium citrate tribasic [(NH_4_)_3_C_6_H_5_O_7_, Beijing TongGuang Fine Chemicals], lithium citrate tribasic (Li_3_C_6_H_5_O_7_, Macklin), sodium sulfate (Na_2_SO_4_, Sinopharm Chemical), ammonium sulfate [(NH_4_)_2_SO_4_, Sinopharm Chemical], lithium sulfate monohydrate (Li_2_SO_4_·H_2_O, J&K), and sodium thiocyanate (NaSCN, Sigma-Aldrich) were used as received.

### Preparation of ssDNA-SWCNT dispersions and PEG/salt ATP solutions

DNA-SWCNT dispersions were prepared according to the previously published procedures (*42*, *45*). Briefly, as-received SWCNT powders in water (1 mg/ml) were mixed with aqueous DNA and salt solutions then dispersed by tip sonication. The typical SWCNT/DNA mass ratio was 1:2.5 for SG65i SWCNTs, and the salt (NaCl) concentration was 30 mM. All percentages referring to the chemical concentration in aqueous solutions were reported on a mass/mass basis.

The 40% PEG6k solution and salt solutions with concentration as high as possible (20 to 40%) need to be prepared to allow the preparation of ATP solutions. For each ATP system, the operating condition was explored according to the previous established method (*42*). ATP stock solutions labeled 10:0 and 7:3, corresponding to the actual operating solution for sorting and the concentrated solution for loading SWCNT dispersion, were prepared before sorting procedures. The top and bottom phases of the 10:0 stock solutions were stored separately when phase separation was completed. They were used as the blank top and bottom phases in the subsequent sorting procedures. The compositions of the 10:0 stock solutions used in this work are listed in Table 1 and table S1. In a typical sorting experiment, 3 volumes (typically 60 μl) of SWCNT dispersion was loaded to 7 volumes (typically 140 μl) of the 7:3 stock solution. After vortex mixing and gentle centrifugation, the first top fraction (1T) was extracted out. After supplementing blank top phase, vortex mixing, and gentle centrifugation, a new top phase was formed and extracted. This process can be repeated multiple times as necessary to achieve the desired separation goal. The detailed sorting process was given in the Supplementary Materials. All experiments related to the aqueous two-phase systems were carried out at 20°C.

### UV-vis-NIR, NIR fluorescence, and CD measurement

UV-vis-NIR absorption spectra were collected on Cary 5000 spectrophotometer or PerkinElmer LAMDA 750 over the wavelength range of 200 to 1400 nm in 1-nm increments using a 10-mm path length quartz microcuvette. Typically, each fraction obtained during the separation was diluted by a factor of 10 before measurement. Blank top and bottom phases diluted by a factor of 10 were used for baseline correction (i.e., for removing the contributions of the cuvette, water, and polymer-salt mixture from the absorbance signal).

PL spectra were collected on a HORIBA Jobin Yvon Nanolog-3 spectrofluorometer. Excitation was selected with a 450-W xenon lamp and dual 1200 by 500 gratings, and emission was collected at 90° and dispersed onto the detector array using a 150 by 1200 grating with an integration time of 10 s. All of the emission spectra were corrected for detector train efficiency and lamp excitation.

CD spectra were recorded with an JASCO J-810 CD spectrophotometer, covering a spectral range of 300 to 850 nm. All of the CD spectra were measured using 1-nm spectral steps and a quartz cell with an optical path length of 1 cm.

### Donnan potential measurement

All the Donnan potential measurements were carried out in a two-electrode system on a CHI 760E electrochemical workstation (Chenhua, China). The electric potentials were recorded as a function of time at an interval of 0.1 s. Before the measurement, each PEG/salt ATP systems (10:0) was prepared according to the recipe listed in Table 1. Those solutions were settled until phase separation was totally completed. Subsequently, the top and bottom phases were carefully extracted and collected separately. The Donnan potential of each PEG/salt ATP system was measured in a U-shaped tube immersed in a 20°C constant temperature water bath, with its right arm filled with the bottom phase of the ATP system and its left arm filled with the corresponding top phase (fig. S28). Two saturated calomel reference electrodes were used using saturated KCl as the primary salt bridge and KCl (0.1 mol/liter) as the secondary salt bridge. The secondary salt bridge was in contact with both the primary salt bridge and the phase where the electrode was introduced through small porous sintered ceramic plugs. The electrodes were rinsed with plenty of ultrapure water and dabbed dry with a tissue before immersion in any solution. Both electrodes and salt bridges were filled to the same height with saturated KCl solution and KCl solution (0.1 mol/liter), respectively, and immersed at the same depth in both phases. The zero potential of the instrument was calibrated by short-circuiting the working electrode, counter electrode, and reference electrode. Then, one electrode was immersed into the top phase in the left arm, while another one was immersed into the bottom phase in the right arm at the same height. The left and right electrodes were used as working electrode and reference electrode, respectively. Open-circuit potential of the system was recorded until the measured data reached a stable value. Then, we switched the two electrodes, keeping the same setup of the electrode connection and implemented the measurement again. By averaging the stable open-circuit potential values obtained from the two measurements, the Donnan potential of the system can be obtained, and the influence of electrode asymmetry can be eliminated. The room temperature was also controlled at 20°C throughout the measurement.

### Photoluminescence quenching profile of 65ss-SWCNTs in salt medium

In general, 65ss-SWCNT dispersion was diluted by 500 times by a specific salt solution with a concentration similar to its concentration in the corresponding PEG/salt ATP systems, which was selected to be 0.6 mol/liter for phosphate salt and 0.42 mol/liter for citrate salt. NaPS and NaClO were used as the oxidant for quenching the photoluminescence (PL) of 65ss-SWCNTs in phosphate salt systems and citrate salt systems respectively due to the following reasons: (i) Quenching in solution containing phosphate salt was done by adding NaPS instead of adding NaClO, because in solution containing ammonium ions, ClO^−^ will undergo complex redox reaction with NH_4_^+^, which prevents the oxidation of SWCNTs. (ii) Oxidation by NaPS is not applicable in citrate systems. The amount of persulfate required to oxidize SWCNTs in the citrate system is much higher than that in the phosphate system. In particular, this will lead to the failure of SWCNT oxidation in the solution containing potassium citrate. Because of the low solubility (≈5 g/100 ml, 20°C) of potassium persulfate, white precipitate of potassium persulfate will be formed after a large amount of NaPS is added to a solution containing potassium citrate.

The PL spectra of 65ss-SWCNTs were measured before the addition of oxidant and then right after each addition of oxidant during the process of quenching experiment. Because the concentration of SWCNTs is in the range where the PL intensity changes linearly with its concentration (*81*, *82*), the PL spectra were calibrated according to the change in SWCNT concentration caused by the volume change with the addition of oxidants. The excitation wavelength was set at 570 nm with the emission spectrum being captured with a center wavelength of 1100 nm.
