# Supplementary material for: A salt-driven mechanism for precise chirality sorting of carbon nanotubes
Source: Sci Adv. 2025 Jul 11;11(28):eadx3958. doi: 10.1126/sciadv.adx3958 (PMC12248291; doi:10.1126/sciadv.adx3958)
Supplement: Supplementary file 1 — Supplementary Text Figs. S1 to S35 Tables S1 to S3 [file sciadv.adx3958_sm.pdf]

Science Advances
AAAS

Supplementary Materials for

A salt-driven mechanism for precise chirality sorting of carbon nanotubes

Min Lyu *et al.*

Corresponding author: Yan Li, yanli@pku.edu.cn; Ming Zheng, ming.zheng@nist.gov

Sci. Adv. **11**, eadx3958 (2025)
DOI: 10.1126/sciadv.adx3958

This PDF file includes:

Supplementary Text
Figs. S1 to S35
Tables S1 to S3

1. The exploration of ATP systems and the meaning of 10:0 and 7:3

Fig. S1A exhibits a schematic phase diagram of aqueous two-phase (ATP) system formed by polyethylene glycol (PEG), salt and water. At low concentrations of polymer and salt, phase separation will not take place and there is only one phase (purple area) in the solution. Phase separation (white area above the purple area) occurs when the concentrations of PEG and salt are above certain levels. The boundary between the single-phase and two-phase region is the binodal or coexistence curve. The hollow dot K on the curve is the critical point and the composition represented by K is the critical composition. For an ATP system whose composition is represented by point A, its top and bottom composition are represented by point T and B and its top to bottom volume ratio equals AB/AT. The line that connects B and T is called the tie line. All the points (such as point A, A' and A'') on the same tie line will have the same top phase and bottom phase except that their top to bottom volume ratios are different.

High concentration PEG and salt solutions are prepared prior to the exploration. Given the above knowledge of phase diagrams, one can explore an ATP system without knowing its phase diagram. As shown in Fig. S1A, upon mixing high concentration PEG and salt solutions (A), by continuously diluting (gray dash line: A-A_1, A_2-A_3) and supplementing the corresponding polymer (green arrow: A_1-A_2) or salt solution (blue arrow: A_3-A_4) to control the volume ratio of top to bottom phase to be close to 1:1, we can approach the critical point of the system.

The experimental process mainly involves two concentrations of ATP solutions, marked as 7:3 and 10:0. The ATP (10:0) is a condition explored to be very close to the critical point, and the ATP (7:3) is a condition with higher polymer and salt concentrations deduced from 10:0 (Fig. S1B).

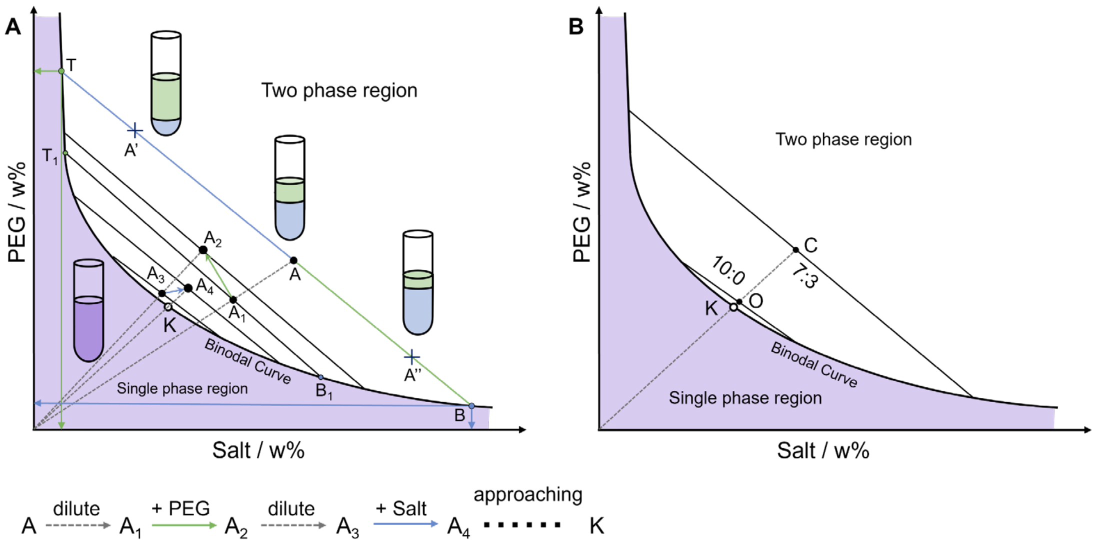

Fig. S1. Schematic phase diagram of an ATP system formed of PEG, salt and water. Schemes illustrating (**A**) the exploration of PEG/salt ATP systems and (**B**) the meaning of 10:0 and 7:3 based on phase diagram.

2. Typical absorbance spectrum and photoluminescence 2D excitation−emission map of the parent DNA-SWCNT dispersion

Below are the absorbance spectrum and photoluminescence 2D excitation−emission map of the parent 65ss-SWCNT dispersion, both revealing a certain degree of (6,5) enrichment while also

indicating the presence of large amounts of other chiral species (such as (8,3), (7,5), (8,4), etc.) within the initial dispersion.

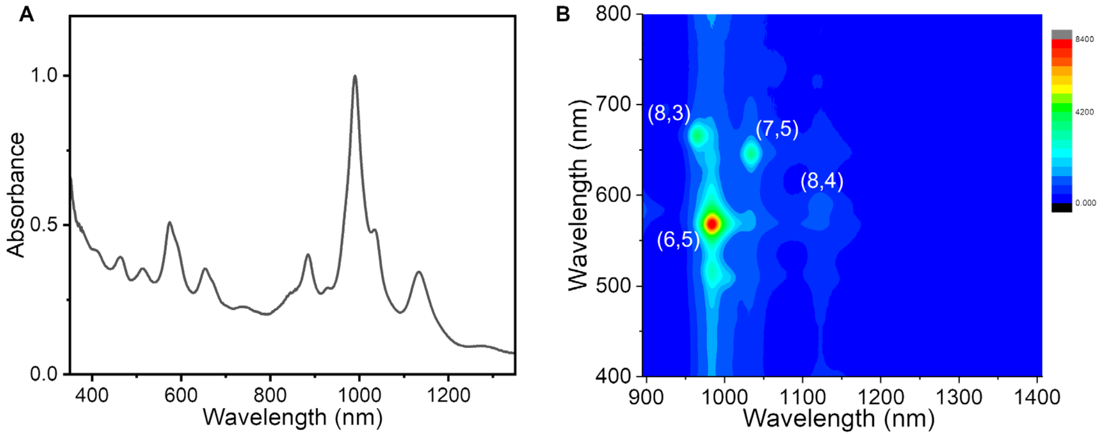

Fig. S2. Typical absorbance and PL spectra of a DNA-SWCNT dispersion prior to sorting. (**A**) Absorbance spectrum and (**B**) photoluminescence 2D excitation−emission map of the parent 65ss-SWCNT dispersion

3. Spontaneous partition of 83ss-SWCNTs in PEG/potassium phosphate and PEG/sodium phosphate ATP systems

For the sorting of 83ss-SWCNTs in the PEG/potassium phosphate ATP system, after phase separation, (8,3)-enriched SWCNTs spontaneously partition to the top phase (1T, absorption spectrum shown as the black curve in Fig. S3A). Similar partition behavior happens throughout the sorting process while more SWCNTs are trapped by the interface compared to the case of 65ss-SWCNTs. Finally, SWCNTs remaining in the bottom phase show some (6,5)-enrichment. For the sorting of 83ss-SWCNTs in the PEG/sodium phosphate ATP system, all SWCNTs partition to the bottom phase and no SWCNTs partition to the top phase (1T, absorption spectrum shown as the black curve in Fig. S3B). There is no tendency to partition to the top phase for SWCNTs after the extraction of top phase and addition of blank top phase. Most SWCNTs are trapped by the interface and precipitated at the bottom of the centrifuge tube and SWCNTs remaining in the bottom phase appears to have no obvious structural enrichment.

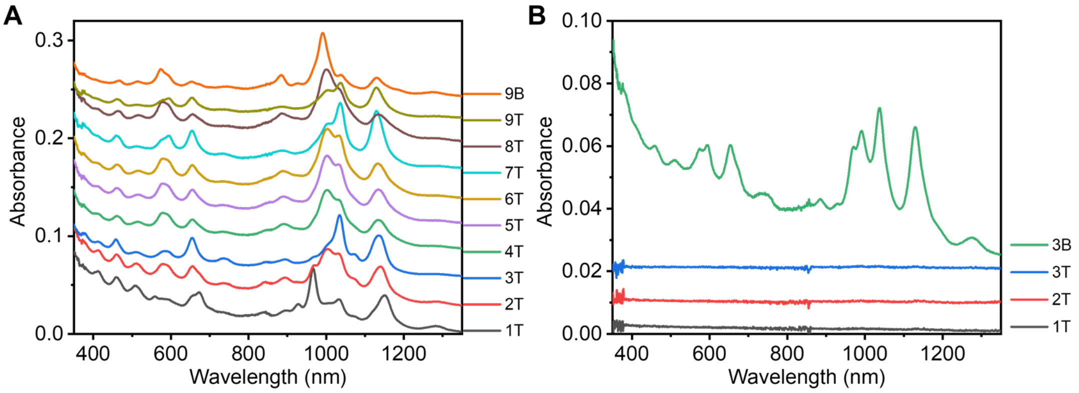

Fig. S3. Spontaneous partition of 83ss-SWCNTs in PEG/salt ATP systems. Absorption spectra of each fraction obtained during the sorting of 83ss-SWCNTs in (**A**) PEG/potassium phosphate ATP system and (**B**) PEG/sodium phosphate ATP system.

4. Anion-dependent partition of DNA-SWCNTs in PEG/salt ATP systems

To compare the effect of anions on the partition of DNA-SWCNTs, the partition results in Figs. 1D to 1G can be divided into four groups according to the type of cations as shown in Fig. S4A to S4D. Take the comparison in the PEG/sodium salt ATP system as an example. According to the concentration of DNA-SWCNTs spontaneously distributed to the top phase (1T) as determined by absorption spectra (Fig. S4A), PEG/sodium citrate is the highest, followed by PEG/sodium sulfate and PEG/sodium phosphate, with PEG/sodium tartrate in the last place. Thus, the modulation capability of anions on the distribution of DNA-SWCNTs is arranged as citrate > sulfate > phosphate > tartrate. Unlike the case for cations, the modulation capability of anions on the distribution of DNA-SWCNTs are not consistent across the four groups.

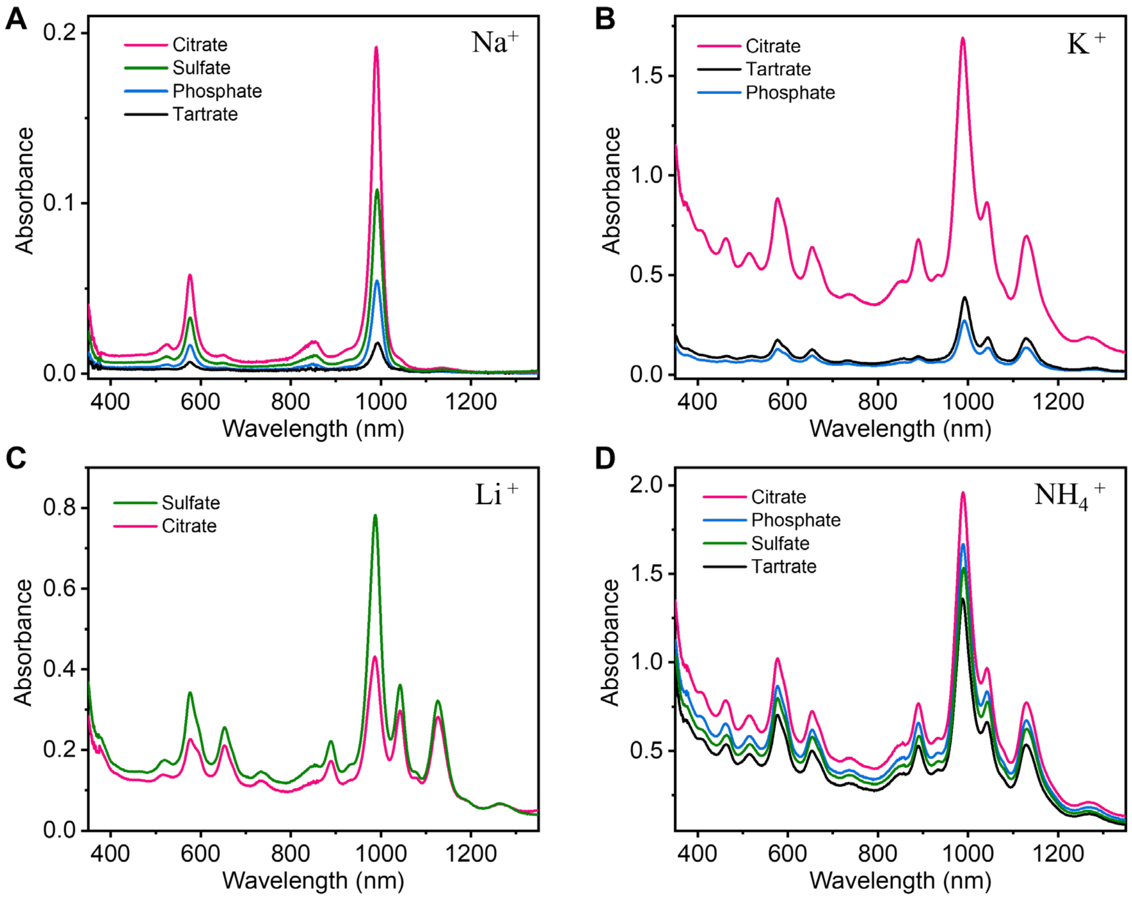

Fig. S4. Anion-dependent partition of DNA-SWCNTs in PEG/salt ATP systems. Absorbance spectra of the first top fraction spontaneously partition to the top phase after loading 65ss-SWCNTs into (**A**) PEG/sodium phosphate, PEG/sodium citrate, PEG/sodium tartrate and PEG/sodium sulfate ATP systems; (**B**) PEG/potassium phosphate, PEG/potassium citrate and PEG/potassium tartrate ATP systems; (**C**) PEG/lithium citrate and PEG/lithium sulfate ATP systems; (**D**) PEG/ammonium phosphate, PEG/ammonium citrate, PEG/ammonium tartrate and PEG/ammonium sulfate ATP systems.

5. Sorting in the PEG/KNa tartrate ATP system

After the first separation step of 65ss-SWCNTs in the PEG/KNa tartrate (Rochelle salt) ATP system, highly-enriched (6,5) (absorbance spectrum shown in Fig. S5) spontaneously partition to

the top phase, while obvious transition peaks contributed by (7,5) as contaminant can still be distinguished.

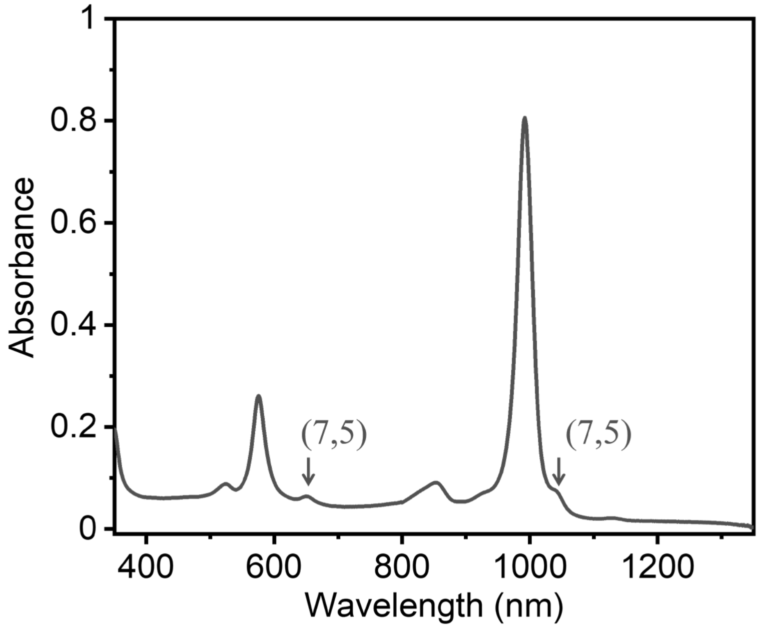

Fig. S5. Spontaneously partition of SWCNTs in the PEG/KNa tartrate ATP system. Absorbance spectrum of the 1T after loading 65sss-SWCNTs in PEG/KNa tartrate ATP system.

6. Composition of PEG/phosphate ATP systems (10:0) with different K/Na ratios used in this work

Table S1. Composition of PEG/phosphate ATP systems (10:0) with different K/Na ratios

		$[K^+]:[Na^+] = 2:1$	$[K^+]:[Na^+] = 1:1$	$[K^+]:[Na^+] = 1:2$	$[K^+]:[Na^+] = 1:3$	$[K^+]:[Na^+] = 1:4$	$[K^+]:[Na^+] = 1:5$	$[K^+]:[Na^+] = 1:6$
	$[K^+]$ / mol·L^{-1}	0.706	0.517	0.330	0.248	0.196	0.160	0.140
PEG6k/Phosphate	$[Na^+]$ / mol·L^{-1}	0.353	0.517	0.660	0.743	0.785	0.798	0.841
	[PEG] / g·L^{-1}	101.677	99.336	94.962	100.326	105.220	102.714	105.220

7. Cation-dependent partition of CTTC$_3$TTC-SWCNTs in PEG/phosphate ATP systems

For CTTC$_3$TTC-SWCNTs, in a series of PEG/phosphate ATP systems with different K/Na ratios, there is an obvious trend of decreasing SWCNT concentration isolated from the top phase (1T) with the increasing ratio of Na^+ in Fig. S6. When KPB is the exclusive component of the phase-forming phosphate salt, (9,1) is a minor species among those SWCNTs spontaneously distributed to the top phase (absorption spectrum shown as the black curve in Fig. S6). With the decrease in the K/Na ratio, SWCNTs other than (9,1) distributed in the top phase dramatically decrease, which means the enrichment of (9,1) is increased. As shown by the absorption spectra in Fig. S6, SWCNTs are extracted with the highest (9,1) enrichment/purity when NaPB is the exclusive component of the phase-forming salt.

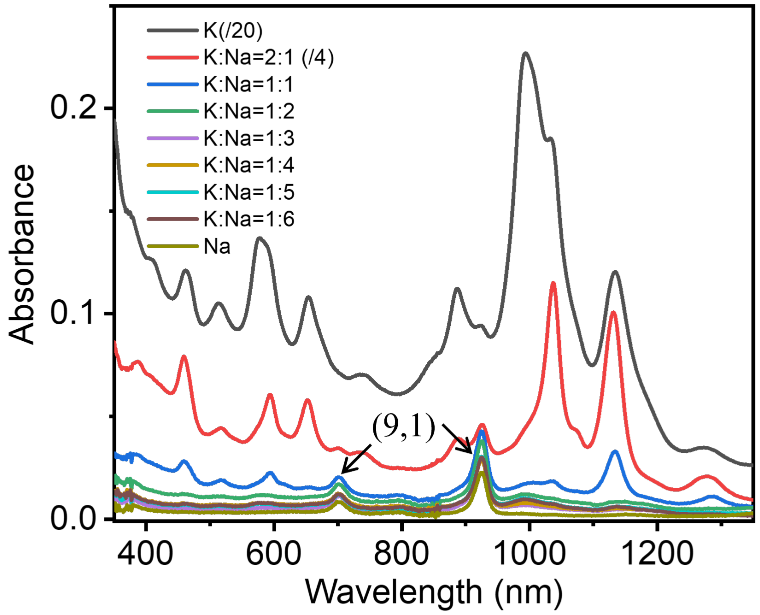

Fig. S6. Optimization of the best K/Na ratio for the single-step extraction of CTTC$_3$TTC-SWCNTs. Absorbance spectra of the SWCNTs spontaneously partition to the top phase after loading CTTC$_3$TTC-SWCNTs into the PEG/Phosphate ATP systems with different K/Na ratios.

8. Spontaneous partition of DNA-SWCNTs in PEG/tartrate and PEG/citrate ATP systems

For 65ss-SWCNTs, in a series of PEG/tartrate and PEG/citrate ATP systems with different K/Na ratios, there is an obvious trend of decreasing SWCNT concentration isolated from the top phase (1T) with the increasing ratio of Na^+ in Fig. S7. When KPB is the exclusive component of the phase-forming phosphate salt, (6,5) along with many other chiral species spontaneously distributed to the top phase (absorption spectra shown as the black curves in Fig. S7A and S7B). With the decrease of K/Na ratio, SWCNTs other than (6,5) distributed in the top phase dramatically decrease, which means the enrichment of (6,5) is increased.

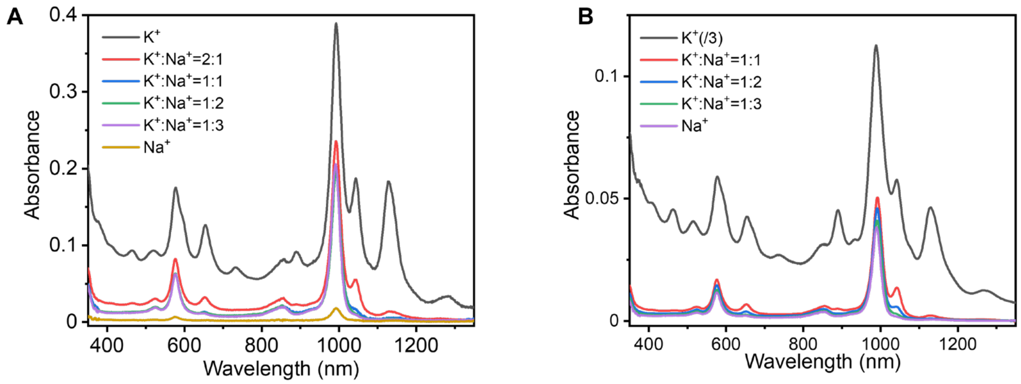

Fig. S7. Optimization of the best K/Na ratio for the single-step extraction of 65ss-SWCNTs. Absorbance spectra of the SWCNTs spontaneously partition to the top phase after loading 65ss-SWCNTs into (**A**) PEG/tartrate and (**B**) PEG/citrate ATP systems with different K/Na ratios.

For 83ss-SWCNTs, in a series of PEG/tartrate ATP systems with different K/Na ratios, there is an obvious trend of decreasing SWCNT concentration isolated from the top phase (1T) with the increasing ratio of Na^+ in Fig. S8. When KPB is the exclusive component of the phase-forming phosphate salt, (8,3) along with many other chiral species spontaneously distributed to the top phase (absorption spectrum shown as the black curve in Fig. S8). With the decrease of K/Na ratio, SWCNTs other than (8,3) distributed in the top phase dramatically decrease, which means the enrichment of (8,3) is increased.

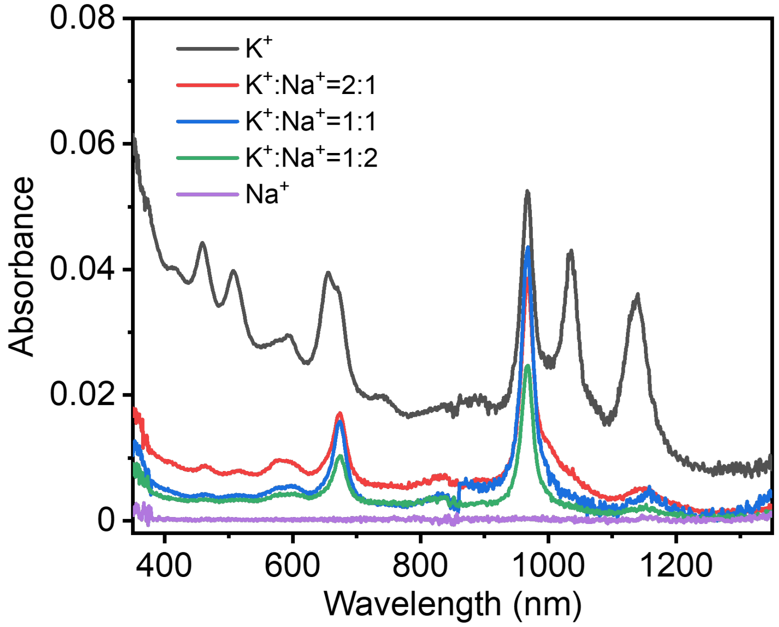

Fig. S8. Optimization of the best K/Na ratio for the single-step extraction of 83ss-SWCNTs. Absorbance spectra of the SWCNTs spontaneously partition to the top phase after loading 83ss-SWCNTs into PEG/tartrate ATP systems with different K/Na ratios.

9. Absorption features appear at 1100 nm to 1200 nm

The absorption spectrum of single-chirality (enantiomer-pure) (6,5) separated from the top phase (1T in Fig. 3C) along with a blank baseline showed an absence of detectable absorption peaks (almost at the blank baseline level as shown by Fig. S9A) within the 1100 nm to1200 nm range.

The absorption spectrum of single-chirality (enantiomer-pure) (6,5) separated from the bottom phase (20B in Fig. 3C) is shown in Fig. S9B (red spectrum). We have also measured the absorbance spectra of DNA (blue spectrum in Fig. S9B) and the PEG/KPB ATP (10:0) (green spectrum in Fig. S9B). Since we use corresponding blank top or bottom phase of ATP (10:0) as the blank baseline for measuring the absorbance of SWCNTs sorted by APTE, the DNA existing in solution and the potential variations of ATP composition due to the salt-switching process may contribute to the absorption feature appears in this region.

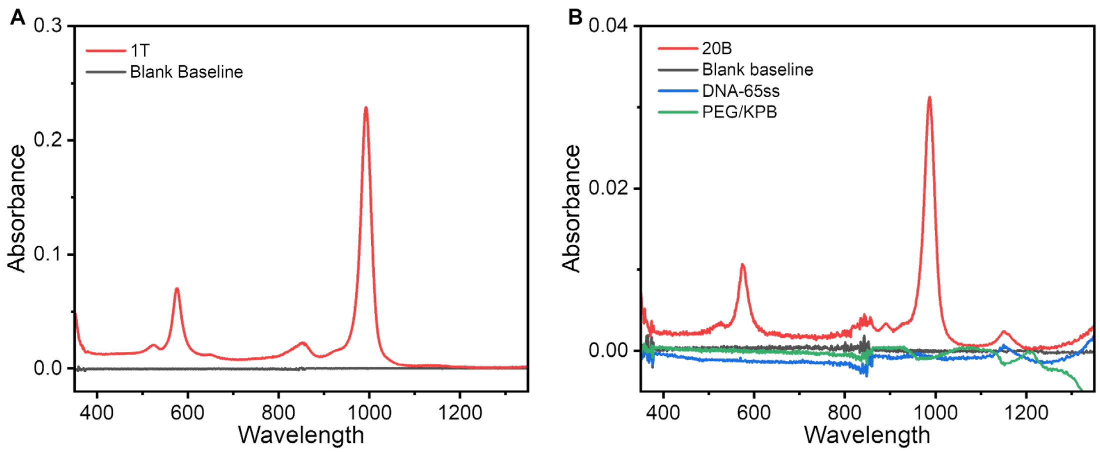

Fig. S9 Absorption features appear at 1100 nm to 1200 nm. (**A**) The absorption spectrum of single-chirality (enantiomer-pure) (6,5) separated from the top phase (1T), along with a blank baseline for comparison. (**B**) The absorption spectrum of single-chirality (enantiomer-pure) (6,5) separated from the bottom phase (20B), along with a blank baseline, absorbance spectra of DNA and PEG/KPB ATP (10:0) for comparison.

10. Batch#2 large-scale extraction of 65ss-SWCNTs

After the extraction of 1T shown in Fig. 3B, blank top phase of PEG/KPB is used to mix with the remaining bottom phase to allow more SWCNTs to partition to the top phase. The blank top phase of PEG/potassium citrate is used after the extraction of 6T and interfacial trapped SWCNTs were removed after the extraction of 7T. With another 7-step extraction, single-chirality (6,5) is also obtained from the bottom phase. Absorption spectra of each fraction obtained during the sorting process are shown in Fig. S10A.

The 1T obtained from two batches of large-scale sorting are collected and precipitated by adding 1.5 mol/L sodium thiocyanate (NaSCN). The nanotube pellet is resuspended in 10 mL water (Fig. S10B). Additionally, a final concentration of 100 μg/mL of the corresponding DNA are added to improve the stability of resuspended DNA-SWCNTs in solution during long-term storage. Single-chirality (6,5) separated from the bottom phase is also redispersed into ~1.6 mL aqueous solution (Fig. S10C) in the same way.

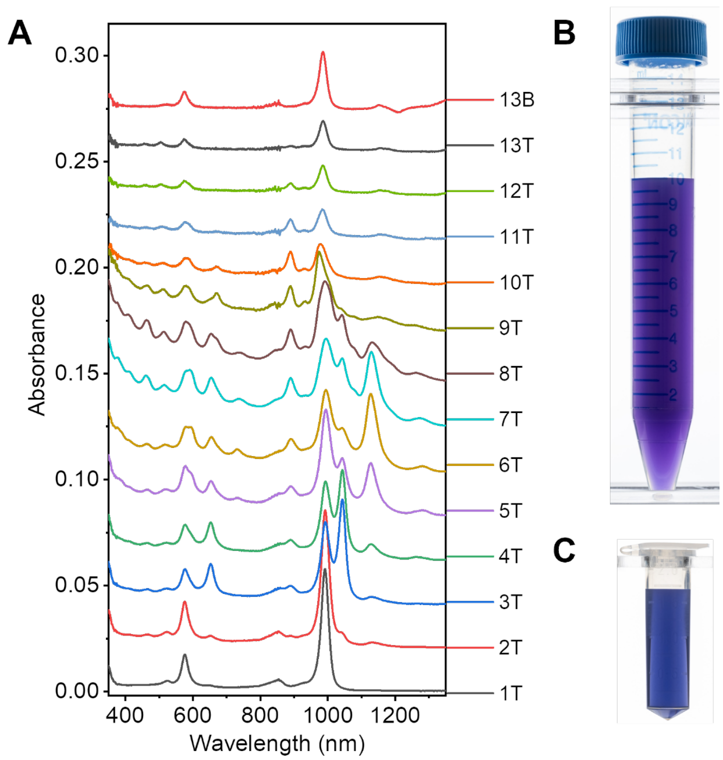

Fig. S10. Batch#2 large-scale extraction of 65ss-SWCNTs. (**A**) Absorption spectra of each fraction obtained throughout the ~40 mL-scale (Batch#2) sorting of 65ss-SWCNTs in PEG/phosphate ATP system with K/Na ratio of 1: 5. (**B**) Single-chirality (6,5) collected from the 1T of two large-scale separation batches then redispersed into ~10 mL aqueous solution. (**C**) Single-chirality (6,5) separated from the bottom phase of two large-scale separation batches then redispersed into ~1.6 mL aqueous solution.

11. Length distributions of SWCNTs in the pristine 65ss-SWCNT dispersion and the sorted (-) (6,5)

Silicon wafers with a thermally grown 500 nm thick SiO_2 layer (denoted as SiO_2/Si) are used for sample deposition. SiO_2/Si substrates are treated with piranha solution at 90 °C for 40 min then immersed in 1% (3-Aminopropyl)triethoxysilane (APTES)/isopropanol (anhydrous) solution for 40 min. The DNA-SWCNT dispersions are diluted to a final concentration of ~10 μg/mL by ultrapure water. APTES-modified SiO_2/Si substrates are soaked in the diluted DNA-SWCNT solutions for 5-10 seconds then rinsed with ultrapure water and dried with nitrogen gas. An AFM (Dimension Icon, Bruker) is used for observations.

1) Pristine 65ss-SWCNT dispersion

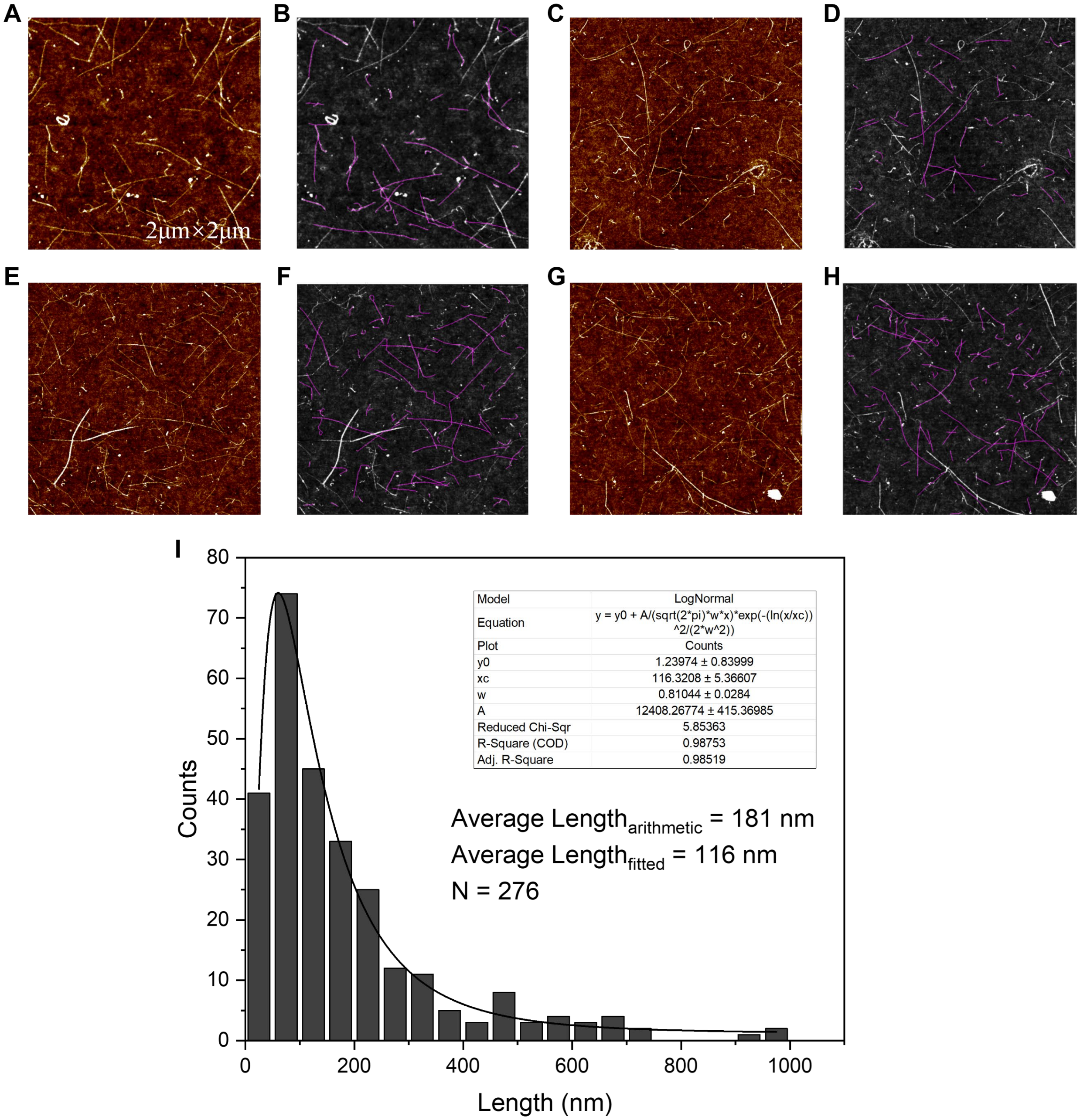

Fig. S11. Length distributions of SWCNTs in the pristine 65ss-SWCNT dispersion. AFM images (**A**, **C**, **E** and **G**) of the deposited SWCNTs from pristine 65ss-SWCNT dispersion at different positions. To the right are grayscale images (**B**, **D**, **F** and **H**) of the AFM images on the left used to count the lengths of the SWCNTs. The SWCNTs highlighted in purple indicate the specific SWCNTs that have been incorporated into the length statistical analysis. (**I**) Length statistics of SWCNTs in pristine 65ss-SWCNT dispersion.

2) (-) (6,5) sorted from top phase

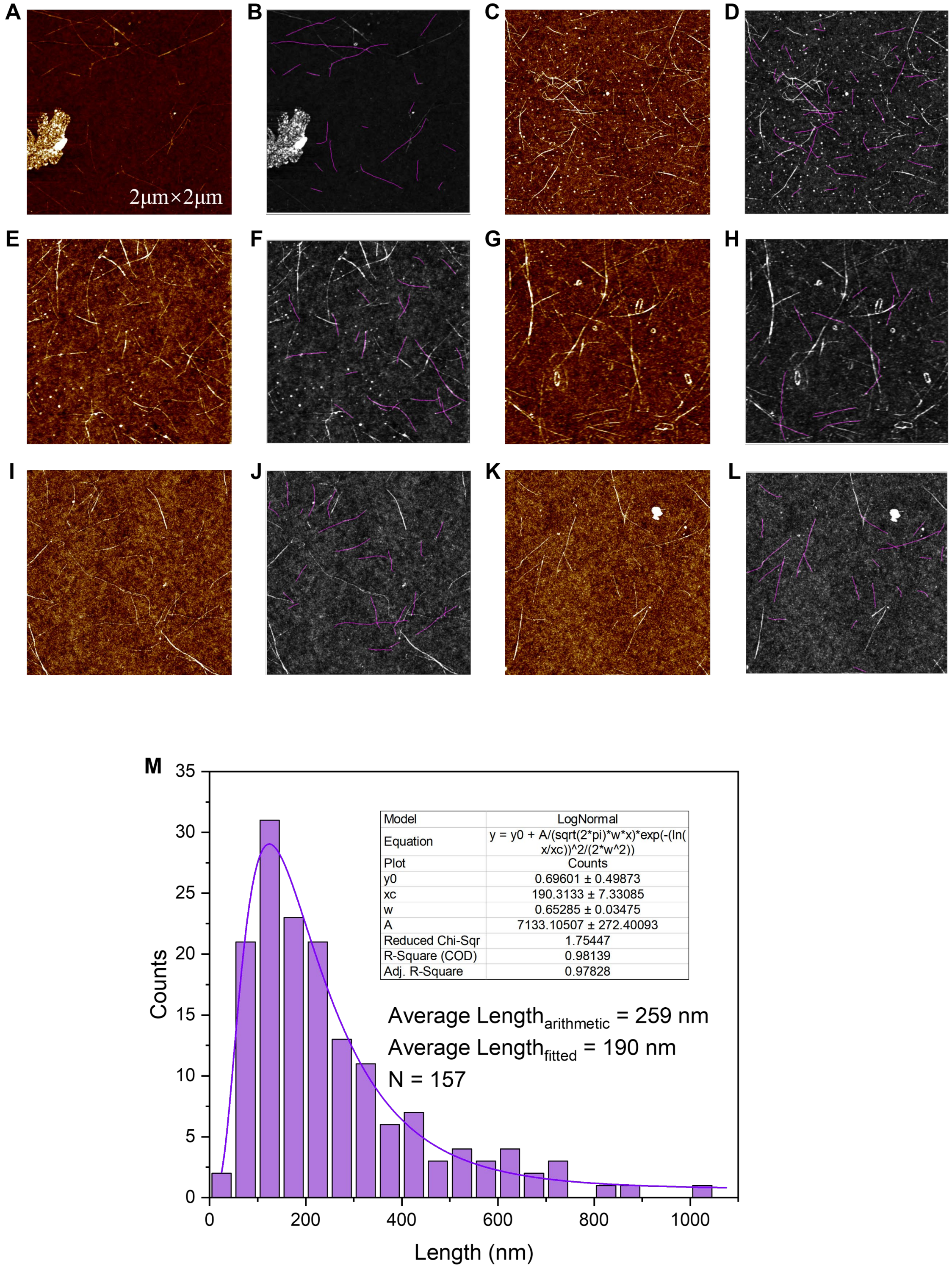

Fig. S12. Length distributions of SWCNTs in the sorted (-) (6,5). AFM images (**A**, **C**, **E**, **G**, **I** and **K**) of the deposited SWCNTs from (-) (6,5) sorted from top phase at different positions. To the right are grayscale images (**B**, **D**, **F**, **H**, **J** and **L**) of the AFM images on the left used to count the lengths of the SWCNTs. The SWCNTs highlighted in purple indicate the specific SWCNTs that have been incorporated into the length statistical analysis. (**M**) Length statistics of (-) (6,5).

12. Analysis of particle length distribution in ATP extraction

ATP extraction may enrich long tubes: For the same (*n,m*), longer tubes have a narrower elution profile than shorter tubes. This is because solvation energy difference of (*n,m*) tubes between the top and bottom phase is proportional to the tube length l: $S_t - S_b \propto l$.

At a given K/Na ratio (or a control parameter for any ATP extraction process) $r = \frac{[K^+]}{[Na^+]}$, the nanotube concentration at top and bottom phase follows $\frac{[n,m]_t}{[n,m]_b} = \exp[S_t - S_b]/kT = \exp[al(r - r_0)/kT]$, where r_0 is the K/Na ratio (or control parameter in general) at which solvation energy in the top and bottom phase is the same, and a is a factor related to the two-phase composition. A broader polymer mass distribution should give a smaller a value, effectively suppressing length dependence shown in the above expression. We can do a numerical analysis below assuming $a = 1$, $kT = 1$ and $r_0 = 0$.

We have two populations of particles: long particles and short particles. The distribution of these particles between the top and bottom phases follows the equation:

[particles in the top phase] / [particles in the bottom phase] = exp($r * l$) , where l is the particle length and r the control parameter.

Given: short particles have a length l = 1; long particles have a length l = L; and the total population of long and short particles is equal and finite.

1) Expression for the Number of Particles in Each Phase

For short particles:

$$\frac{N_{short,top}}{N_{short,bottom}} = e^r$$

For long particles:

$$\frac{N_{long,top}}{N_{long,bottom}} = e^{rL}$$

2) Normalization with Finite Total Population

Assuming the total population of each type of particle is equal and finite:

$$N_{short,top} + N_{short,bottom} = N_{short,total} = N_{long,top} + N_{long,bottom} = N_{long,total}$$

3) Ratio of Long to Short Particles in the Top Phase

$$\frac{N_{long,top}}{N_{short,top}} = \frac{e^{r(L-1)}(1+e^{r})}{1+e^{rL}}$$

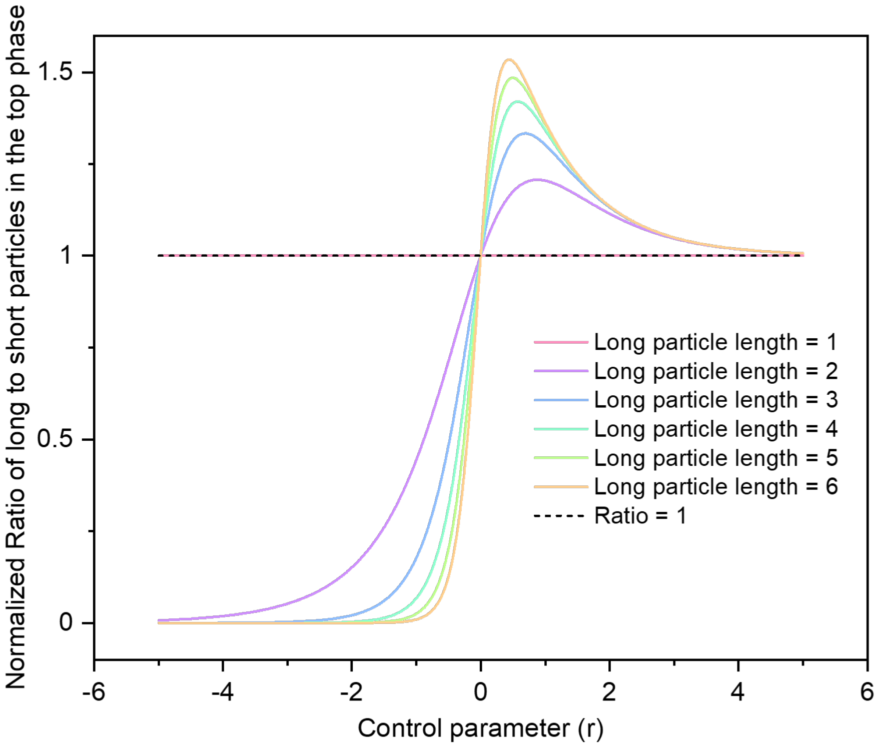

Fig. S13. Normalized ratio of long to short particle population in the top phase as a function of r.

For top-phase extraction, one typically sets r slightly above 0. For bottom-phase extraction, one typically sets r slightly below 0. In both cases, the final extracted phase is enriched in longer tubes.

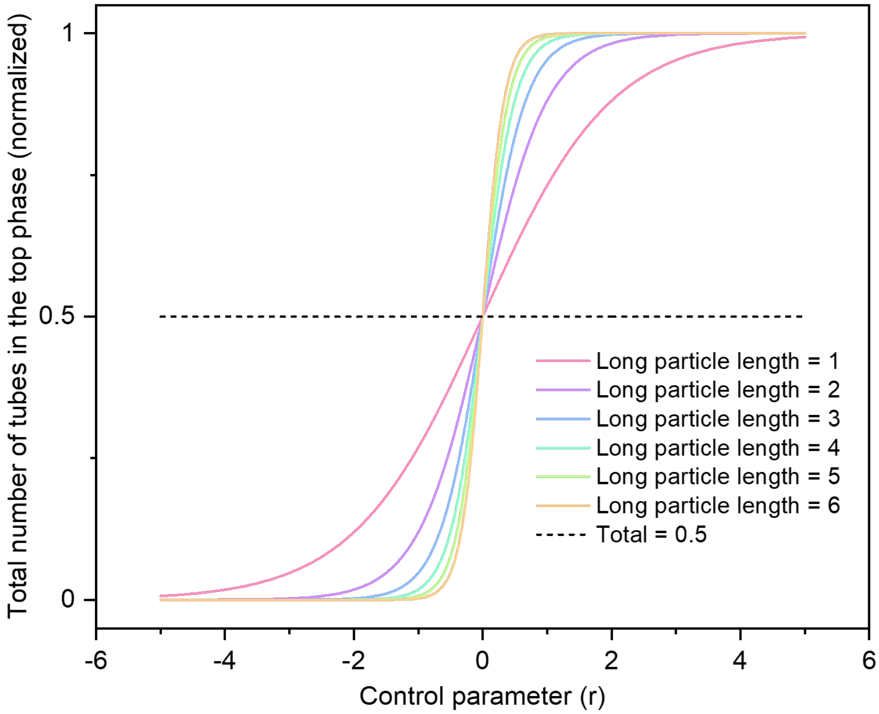

Fig. S14. Total number of tubes in the top phase as a function of r for L= 1 to 6 (Normalized).

$$f\ (r) = \frac{e^{rL}}{1 + e^{rL}}$$

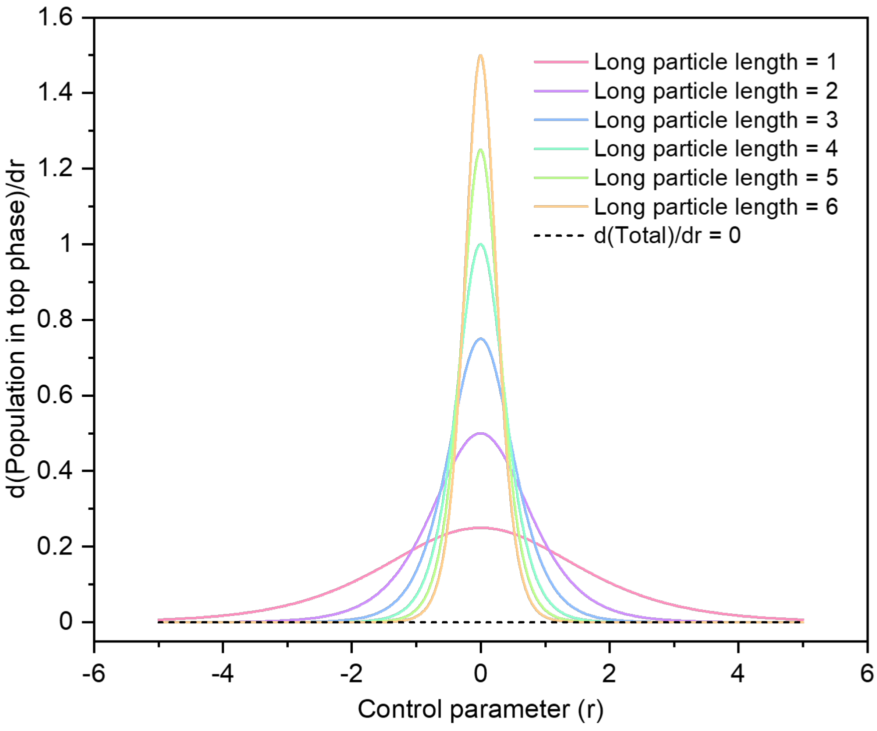

Fig. S15. Derivative of population in top phase with respect to r for long tubes (L = 1 to 6).

$$g(r) = \frac{df(r)}{dr} = \frac{L\, e^{rL}}{(1 + e^{rL})^2}$$

13. SWCNT loss by interfacial trapping

By adding the absorption spectra of all the fractions obtained during multi-stage sorting of 65ss-SWCNTs and 83ss-SWCNTs shown in Fig. 3C and Fig. 3G (red trace in Fig. S16A and Fig. S16B, corresponding to the DNA-SWCNTs recovered during ATPE) and comparing it with the absorption spectra of the initial dispersion (black trace in Fig. S16A and Fig. S16B), it can be roughly concluded that interfacial trapping causes around 26% of the SWCNT loss for 65ss-SWCNT dispersion and 55% of the SWCNT loss for 83ss-SWCNT dispersion. According to their difference (blue trace in Fig. S16A and Fig. S16B, corresponding to the SWCNTs loss caused by interfacial trapping during ATPE), it can be seen that (6,5) also exists in the interfacial-trapped SWCNTs.

Although the mechanism behind the interfacial trapping phenomenon is not fully understood, our previous observations suggest that the interfacial trapping may be accounted for by the depletion force based mechanism (*67*). The active concentration of water is markedly decreased as the concentration of salt is very high in PEG/salt ATP systems. Molecular-crowding-induced clustering may accelerate the formation of SWCNT aggregation or bundles, especially for DNA-SWCNT dispersions prepared with DNA sequences that do not interact strongly with SWCNTs. These aggregated or bundled DNA-SWCNTs may be trapped by the interface of ATP system which is the interfacial trapping we observed.

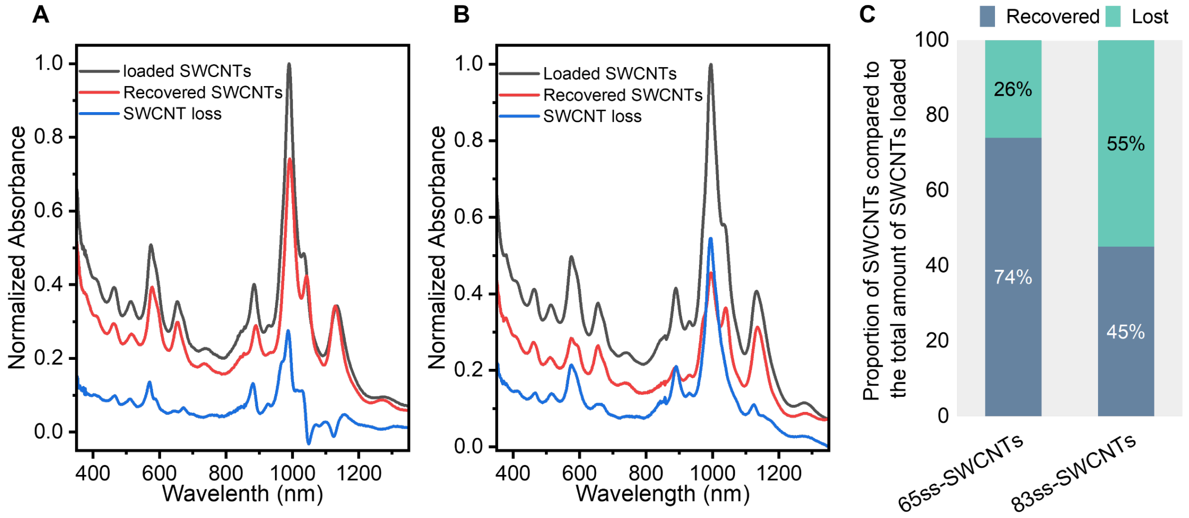

Fig. S16. SWCNT loss during multistage sorting. Normalized absorbance spectra of the total amount of SWCNTs loaded into the ATP system, recovered SWCNTs and SWCNT loss during the multistage sorting of (**A**) 65ss-SWCNTs and (**B**) 83ss-SWCNTs. (**C**) The proportion of recovered SWCNTs and lost SWCNTs compared to the total amount of SWCNTs loaded into the ATP system.

14. The adverse effects of 83ss-SWCNT instability on (8,3) separation purity in PEG/sulfate aqueous two-phase systems

In sulfate salt systems, which is another inorganic salt explored in this work, the concept of optimizing cation composition (NH_4^+/Na^+ or Li^+/Na^+ ratios) also works since the modulation capability of cations on the distribution of DNA-SWCNTs in sulfate salt system is arranged as $NH_4^+ > Li^+ > Na^+$. As shown in Fig. S17A, for 83ss-SWCNT dispersion, compared to the

PEG/Na_2SO_4 ATP system, when Li^+: Na^+= 1 or NH_4^+: Na^+= 1, more SWCNTs spontaneously partition to the top phase. However, as shown in Fig. S17B- S17C, the purity of (8,3) in the SWCNTs that spontaneously partition to the top phase was not as good as that obtained in the PEG/sodium phosphate APT system and PEG/phosphate system with K^+: Na^+= 1. We noticed that more serious interfacial trapping and precipitation occurred during the sorting in the PEG/sulfate system compare to that in the PEG/phosphate system, which also explains why the purity of the (8,3) we obtained in this system is not as good as that in the PEG/phosphate salt system.

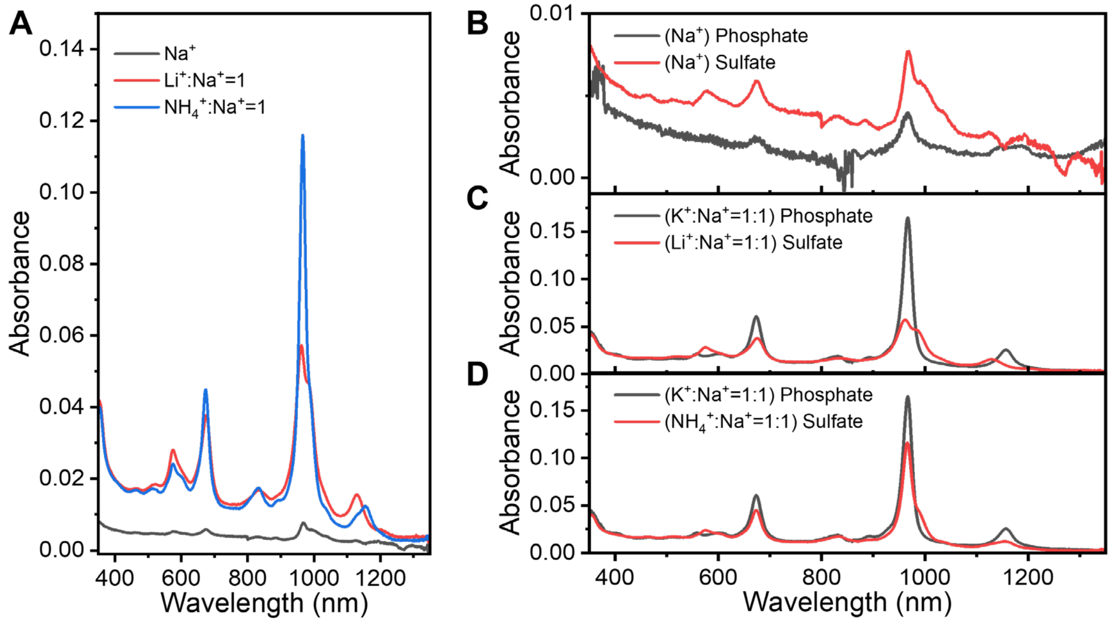

Fig. S17 The sorting of 83ss-SWCNTs in PEG/sulfate ATP systems. (**A**) Absorbance spectra of the DNA-SWCNTs spontaneous partition to the top phase of PEG/sulfate system with different cation composition. Comparison of the absorbance spectra of the DNA-SWCNTs spontaneous partition to the top phase of (**B**) PEG/ sodium phosphate system and PEG/ sodium sulfate system, (**C**) PEG/ (K^+: Na^+= 1) phosphate system and PEG/ (Li^+: Na^+= 1) sulfate system, (**D**) PEG/ (K^+: Na^+= 1) phosphate system and PEG/ (NH_4^+: Na^+= 1) sulfate system.

15. Sorting results and interfacial trapping phenomenon of other tested DNA sequences

Below are the sorting results of SWCNT dispersions prepared with other tested DNA sequences.

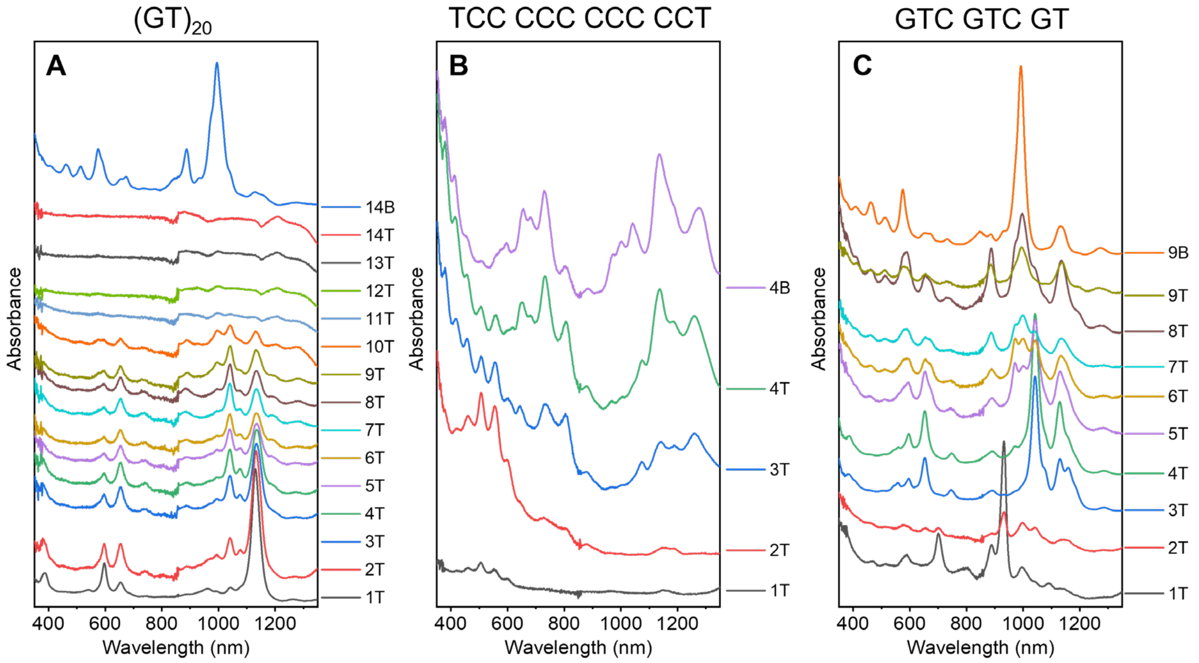

Fig. S18 Sorting outcomes of SWCNT dispersions prepared with $(GT)_{20}$, TCC CCC CCC CCT and GTC GTC GT. Absorption spectra of each fraction obtained during the sorting of (**A**) $(GT)_{20}$-SWCNTs, (**B**) TCC CCC CCC CCT-SWCNTs and (**C**) GTC GTC GT-SWCNTs.

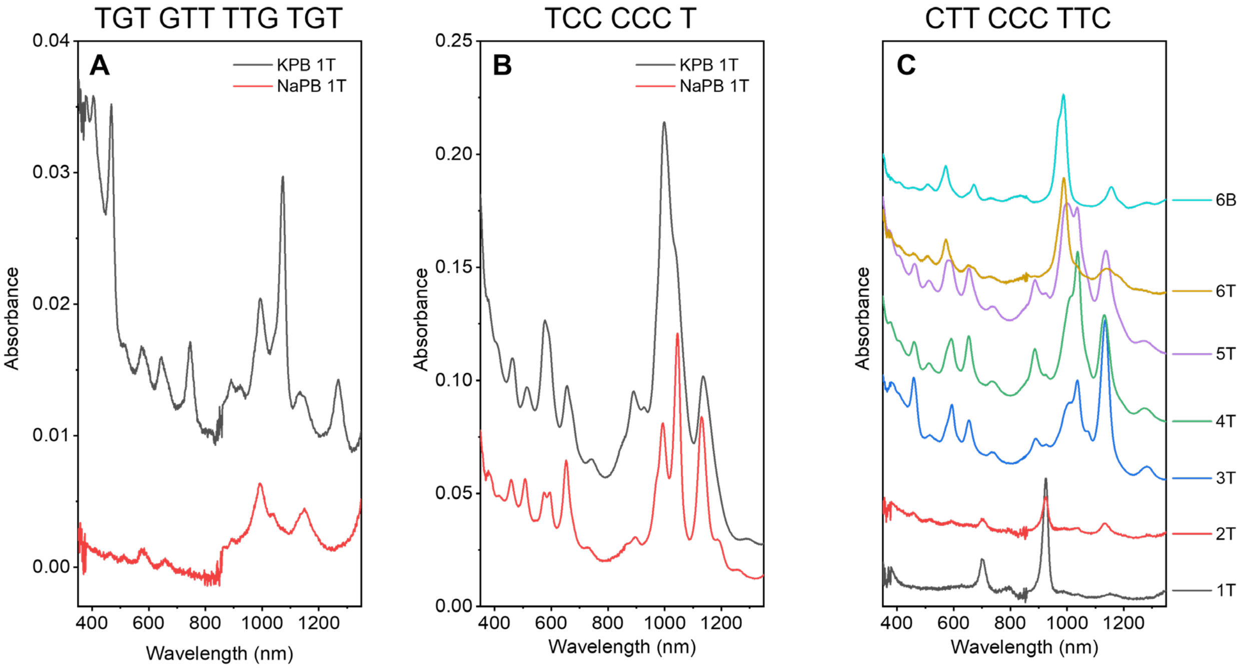

Fig. S19 Sorting outcomes of SWCNT dispersions prepared with TGT GTT TTG TGT, TCC CCC T and CTT CCC TTC. Absorption spectra of each fraction obtained during the sorting of (**A**) TGT GTT TTG TGT-SWCNTs, (**B**) TCC CCC T-SWCNTs and (**C**) CTT CCC TTC-SWCNTs.

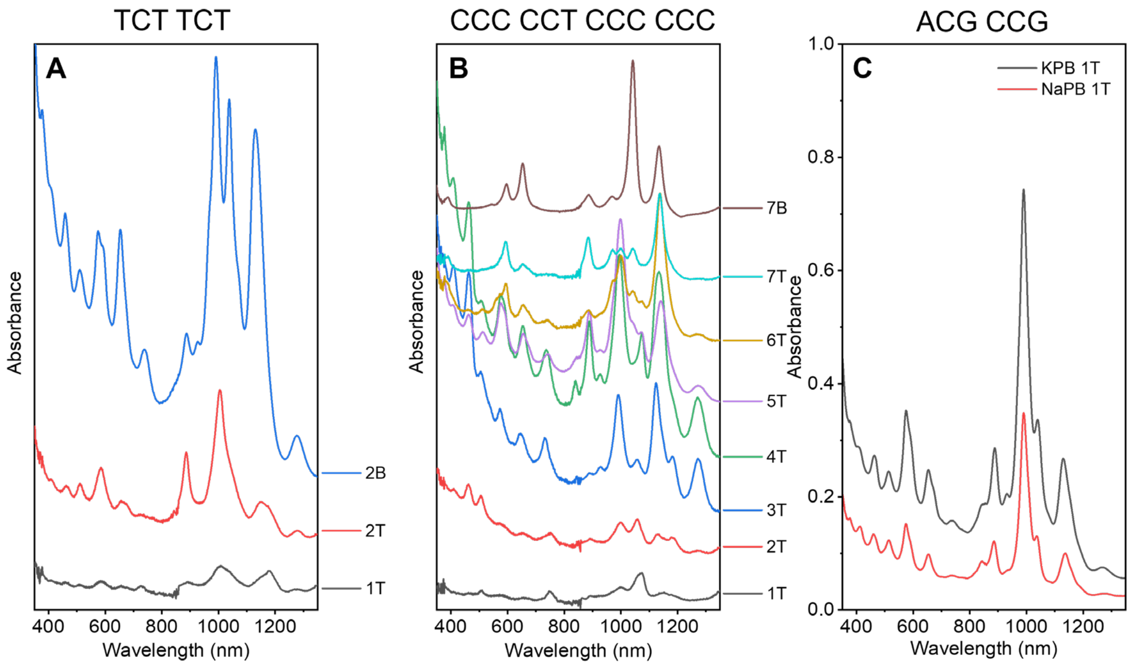

Fig. S20 Sorting outcomes of SWCNT dispersions prepared with TCT TCT, CCC CCT CCC CCC and ACG CCG. Absorption spectra of each fraction obtained during the sorting of (**A**) TCT TCT-SWCNTs, (**B**) CCC CCT CCC CCC-SWCNTs and (**C**) ACG CCG-SWCNTs.

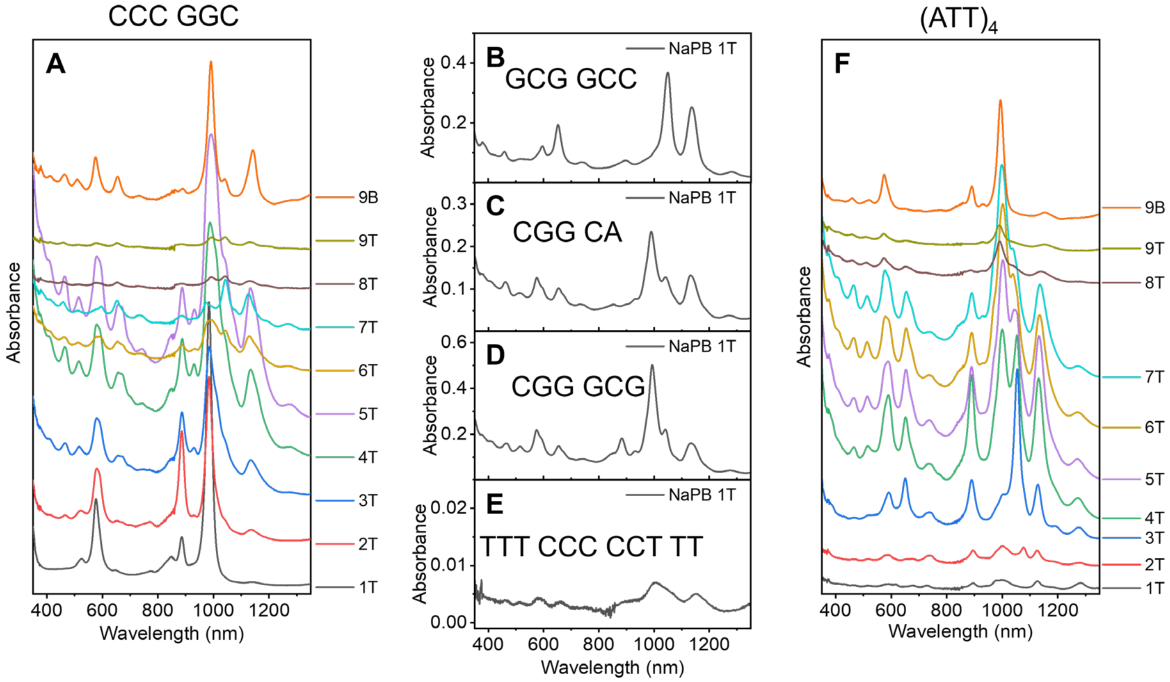

Fig. S21 Sorting outcomes of SWCNT dispersions prepared with CCC GGC, GCG GCC, CGG CA, CGG GCG, TTT CCC CCT TT and $(ATT)_4$. Absorption spectra of each fraction obtained during the sorting of (**A**) CCC GGC-SWCNTs, (**B**) GCG GCC-SWCNTs, (**C**) CGG CA-SWCNTs, (**D**) CGG GCG-SWCNTs, (**E**) TTT CCC CCT TT-SWCNTs and (**F**) $(ATT)_4$-SWCNTs.

The interfacial trapping or precipitation phenomena observed during the sorting process are summarized in Table S2. We did not observe a preferential occurrence of precipitation or severe interfacial trapping for specific base pair combinations. However, our preliminary data suggest that shorter DNA sequences wrapped SWCNTs exhibit a higher propensity for severe interfacial trapping in PEG/salt ATP systems. Given the limited number of sequences tested to date, we anticipate that more definitive patterns may emerge with expanded sequence diversity in future studies.

Table S2 Interfacial trapping phenomena of DNA-SWCNTs

DNA Sequences	Partition Behavior
TTA TAT TAT ATT	slight interfacial trapping
TTT CCC TTT CCC CCC	slight interfacial trapping
$(GT)_{20}$	interfacial trapping and precipitation
TCC CCC CCC CCT	interfacial trapping
GTC GTC GT	interfacial trapping
TGT GTT TTG TGT	multistage sorting not tested, no interfacial trapping at first step
TCC CCC T	multistage sorting not tested, no interfacial trapping at first step
CTT CCC TTC	severe interfacial trapping
TCT TCT	severe interfacial trapping
CCC CCT CCC CCC	interfacial trapping and precipitation
ACG CCG	severe interfacial trapping
CCC GGC	interfacial trapping
GCG GCC	Severe interfacial trapping
CGG CA	All CNT partition to top when using NaPB
CGG GCG	Almost All CNT partition to top when using NaPB
$(ATT)_4$	interfacial trapping
TTT CCC CCT TT	Severe interfacial trapping and precipitation

16. Photoluminescence quenching of 65ss-SWCNTs in salt medium

1) Ammonium phosphate

The concentration NaPS is increased from 0 mmol/L to 1190.1 mmol/L.

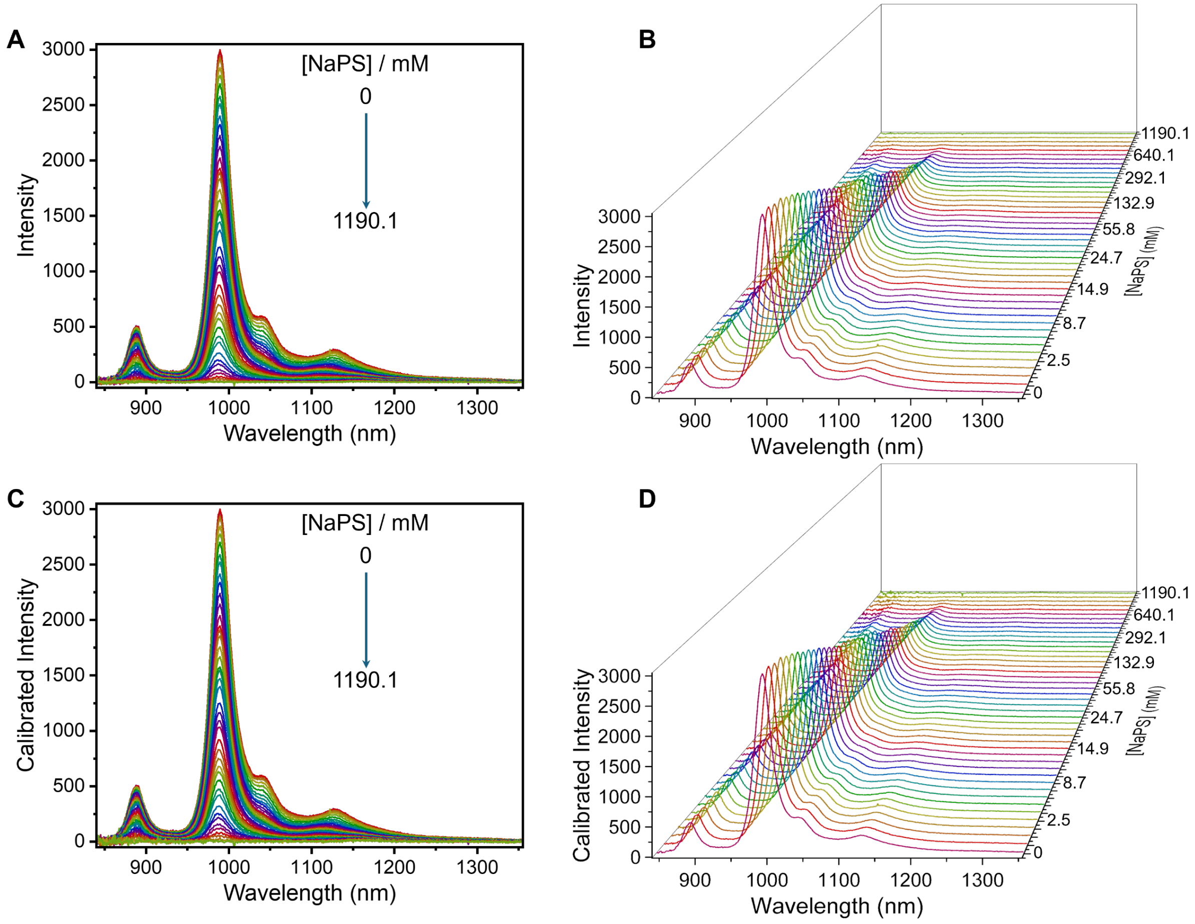

Fig. S22. Photoluminescence quenching of 65ss-SWCNTs in ammonium phosphate solution. Original PL spectra (excitation wavelength: 570 nm) of 65ss-SWCNTs (0.6 mol/L ammonium phosphate) under different sodium persulfate concentration. (**A**) stacked view. (**B**) waterfall view. Calibrated PL spectra (**C**) stacked view. (**D**) waterfall view.

2) Potassium phosphate

The concentration NaPS is increased from 0 mmol/L to 194.8 mmol/L.

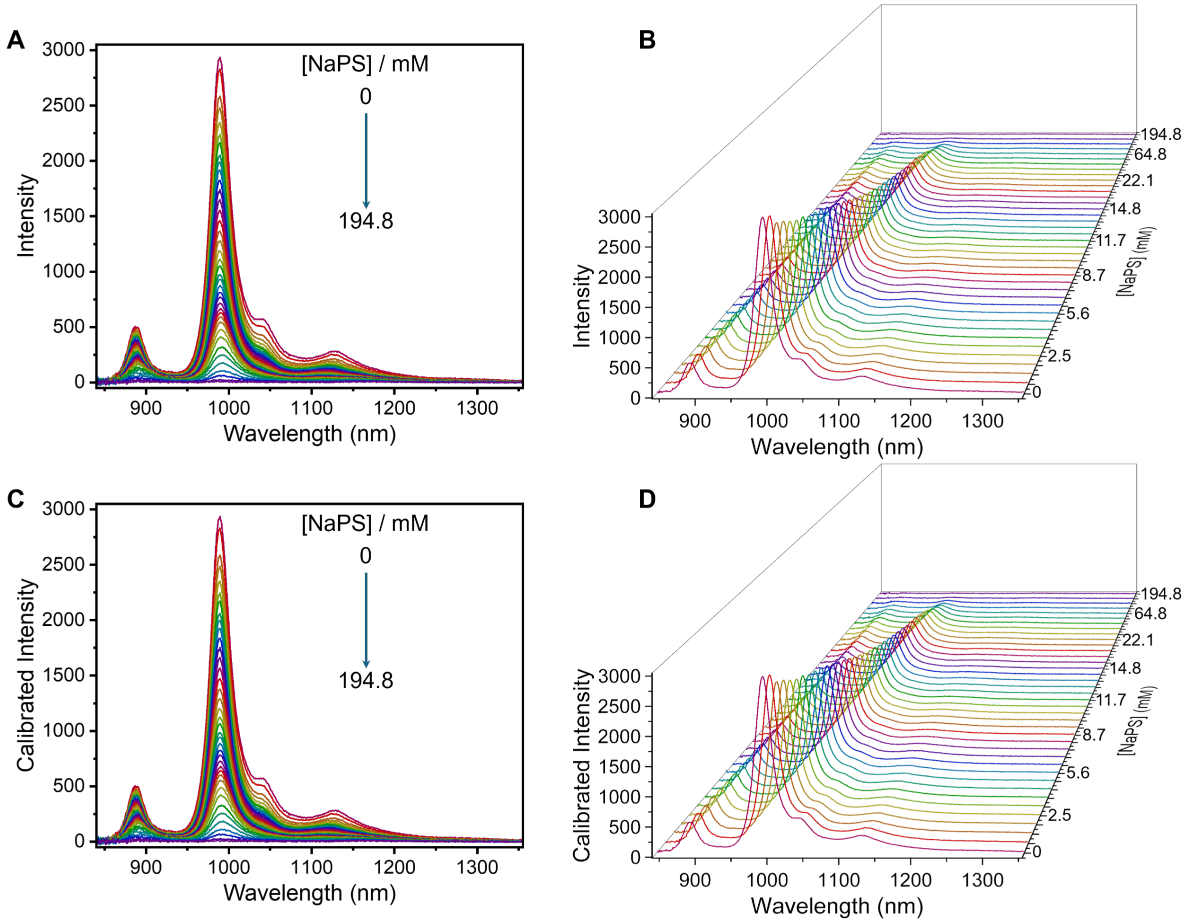

Fig. S23. Photoluminescence quenching of 65ss-SWCNTs in potassium phosphate solution. Original PL spectra (excitation wavelength: 570 nm) of 65ss-SWCNTs (0.6 mol/L potassium phosphate) under different sodium persulfate concentration. (**A**) stacked view. (**B**) waterfall view. Calibrated PL spectra (**C**) stacked view. (**D**) waterfall view.

3) Sodium phosphate

The concentration NaPS is increased from 0 mmol/L to 67.3 mmol/L.

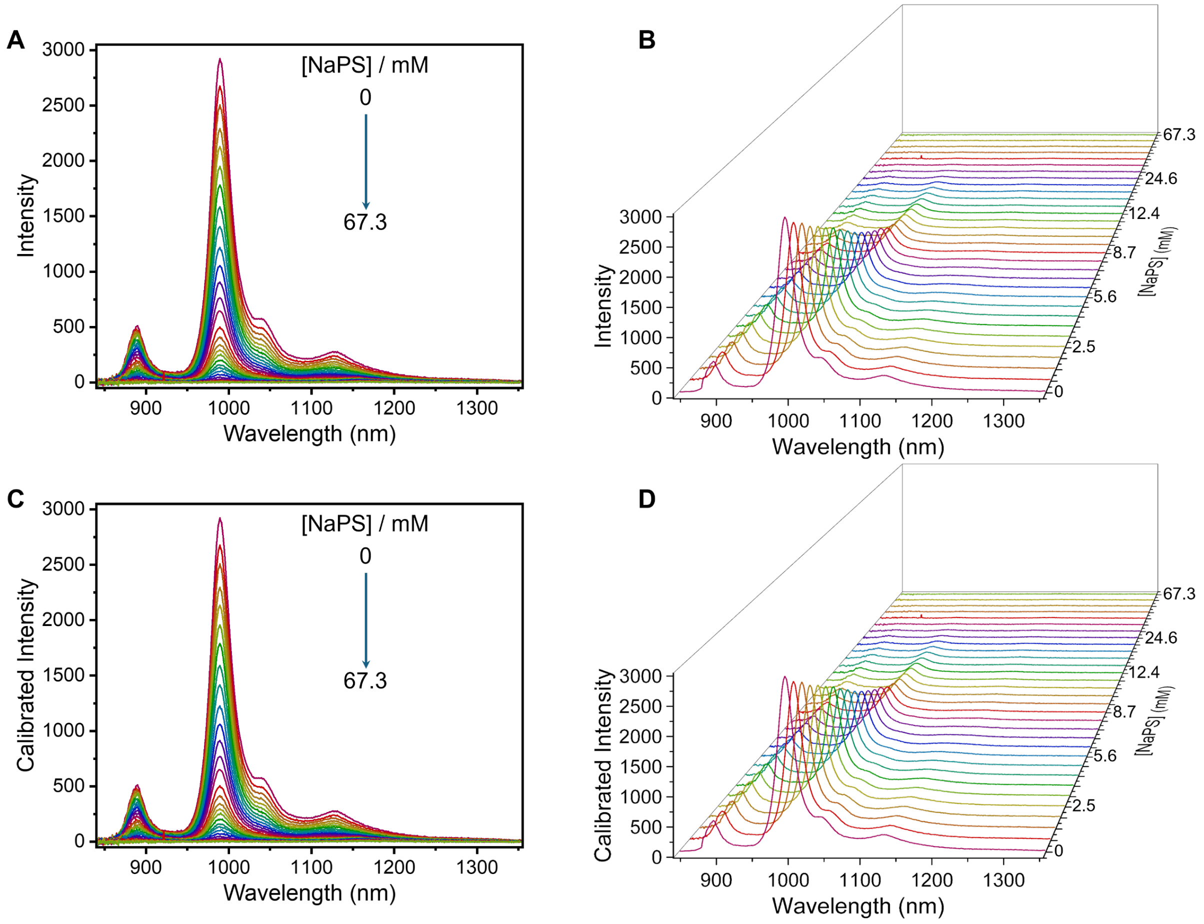

Fig. S24. Photoluminescence quenching of 65ss-SWCNTs in sodium phosphate solution. Original PL spectra (excitation wavelength: 570 nm) of 65ss-SWCNTs (0.6 mol/L sodium phosphate) under different sodium persulfate concentration. (**A**) stacked view. (**B**) waterfall view. Calibrated PL spectra (**C**) stacked view. (**D**) waterfall view.

4) Potassium citrate

The concentration NaClO is increased from 0 μmol/L to 110.9 μmol/L.

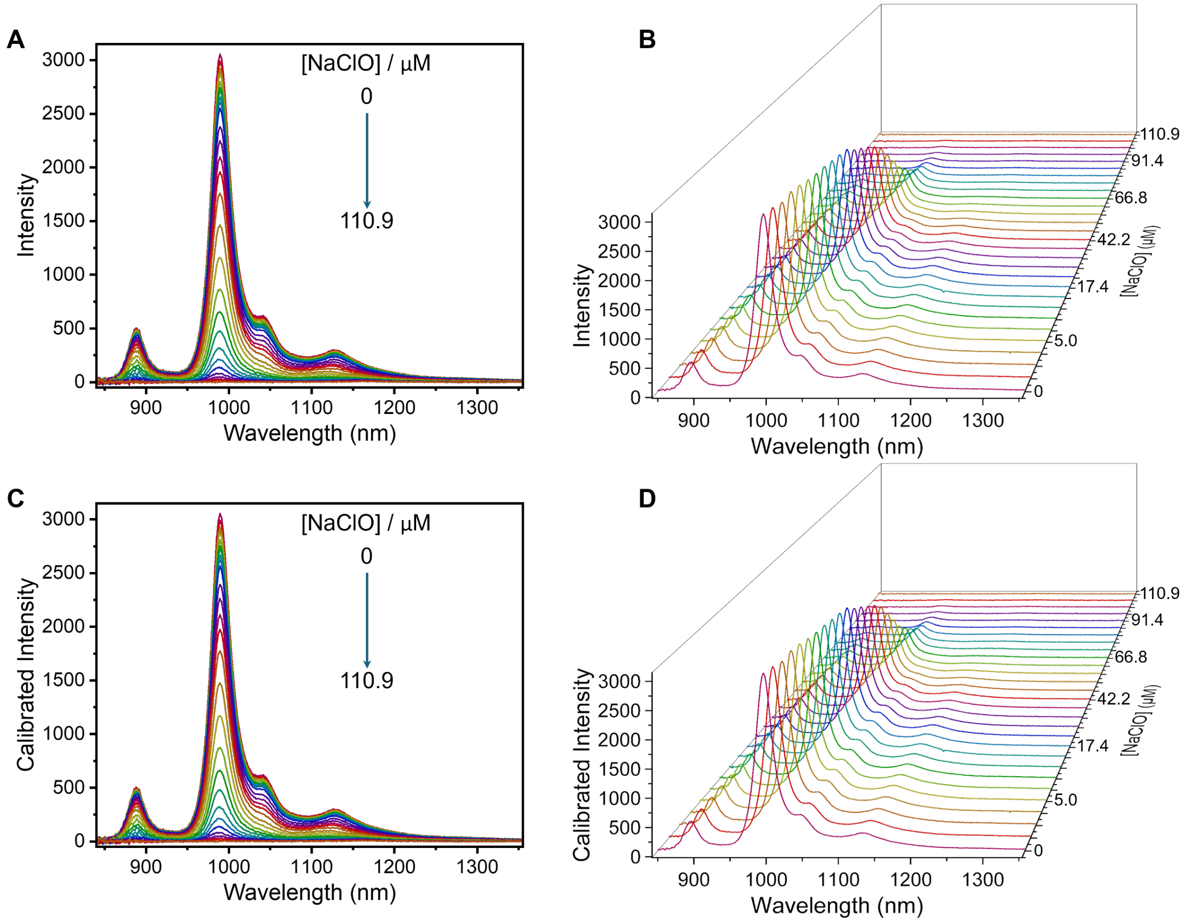

Fig. S25. Photoluminescence quenching of 65ss-SWCNTs in potassium citrate solution. Original PL spectra (excitation wavelength: 570 nm) of 65ss-SWCNTs (0.42 mol/L potassium citrate) under different sodium hypochlorite concentration. (**A**) stacked view. (**B**) waterfall view. Calibrated PL spectra (**C**) stacked view. (**D**) waterfall view.

5) Lithium citrate

The concentration NaClO is increased from 0 μmol/L to 115.5 μmol/L.

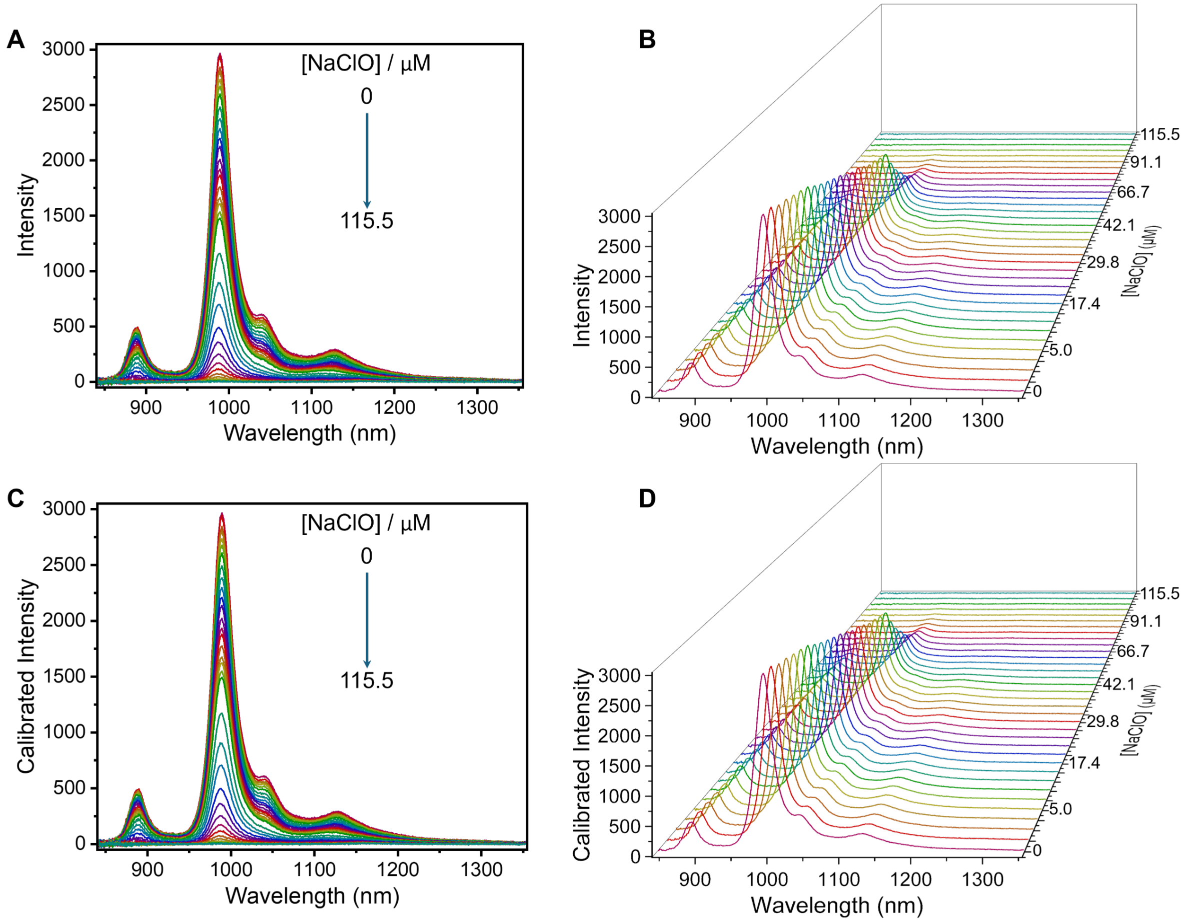

Fig. S26. Photoluminescence quenching of 65ss-SWCNTs in lithium citrate solution. Original PL spectra (excitation wavelength: 570 nm) of 65ss-SWCNTs (0.42 mol/L lithium citrate) under different sodium hypochlorite concentration. (**A**) stacked view. (**B**) waterfall view. Calibrated PL spectra (**C**) stacked view. (**D**) waterfall view.

6) Sodium citrate

The concentration NaClO is increased from 0 μmol/L to 95.8 μmol/L.

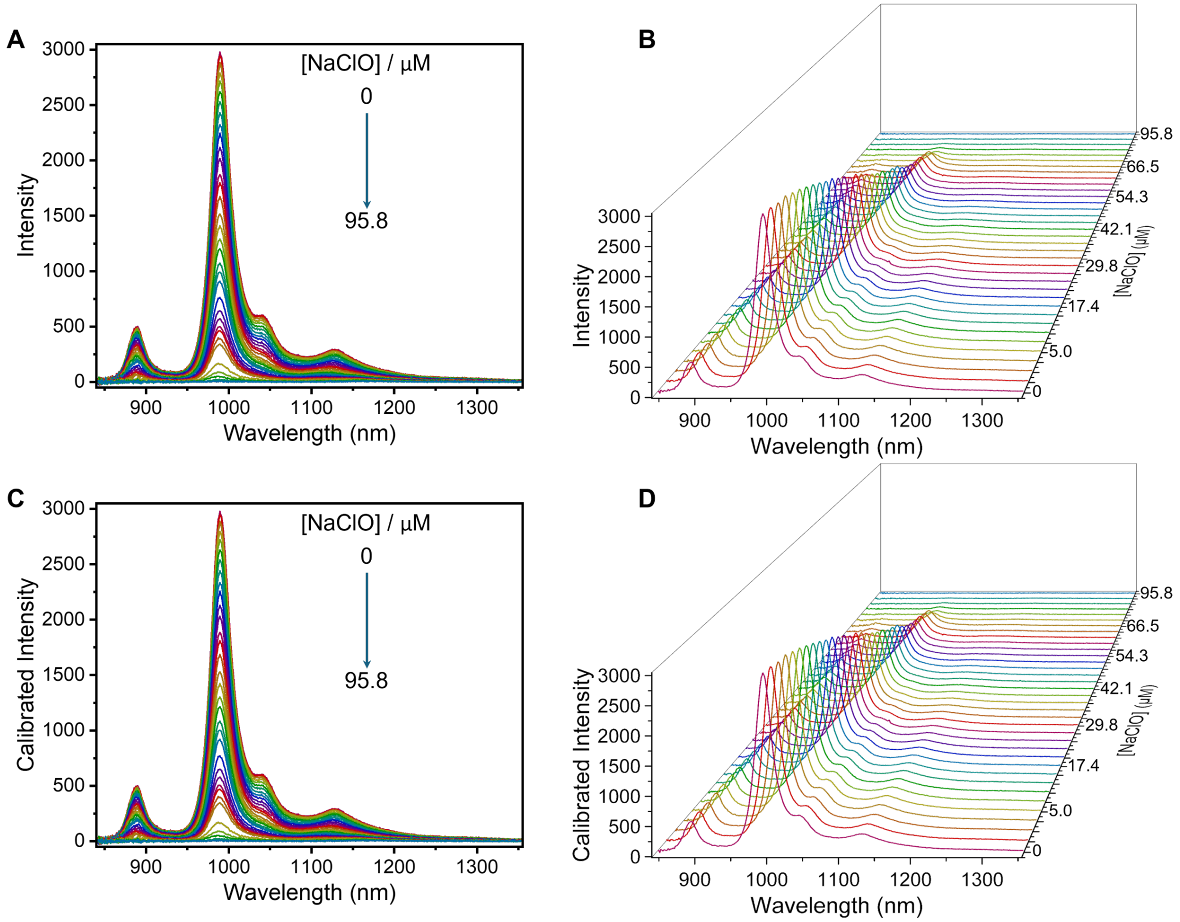

Fig. S27 Photoluminescence quenching of 65ss-SWCNTs in sodium citrate solution. Original PL spectra (excitation wavelength: 570 nm) of 65ss-SWCNTs (0.42 mol/L sodium citrate) under different sodium hypochlorite concentration. (**A**) stacked view. (**B**) waterfall view. Calibrated PL spectra (**C**) stacked view. (**D**) waterfall view.

17. Discussion on the effect of pH on separation

The pH value of all the salt solutions (Table S3) are measured under the same concentration conditions as in the sorting experiment.

Table S3 The pH values of all the salt solutions used in the experiment

Salts		pH
Phosphate	Na	6.63
	K	6.80
	NH_4	8.05
Citrate	Na	8.15
	Li	8.26
	K	8.04
	NH_4	7.03
Tartrate	Na	7.32
	K	7.4
	NH_4	6.49
Sulfate	Na	6.85
	Li	8.29
	NH_4	5.24

Due to the following reasons, we suggest that pH is not the dominant factor affecting separation in this case.

a) While sodium and potassium salts exhibit similar pH values, their modulation capabilities differ markedly**,** strongly suggesting that pH is not the dominant factor in our separation mechanism.

b) Protonation/deprotonation of DNA bases do not occur within the pH range of these salt solutions:

In a previous work reported by us (*42*), we observed that low pH conditions are better for the sorting of zigzag (11,0) in PEG/dextran ATP system when using cytosine (C)-rich DNA sequences. This phenomenon was hypothesized to arise from partial protonation of cytosine bases under low pH conditions, which may promote hydrogen bonding between protonated and non-protonated cytosine residues, thereby enhancing ordered DNA wrapping on zigzag SWCNTs. We have measured the pH value of the salt solution used in this work. The pH ranges of these salt solutions fall outside the protonation/deprotonation thresholds of the nucleobases in our DNA sequences (e.g., Adenine (A) imino nitrogen (N1): pKa $\approx$ 4.2, Cytosine (C) imino nitrogen (N3): pKa $\approx$ 4.5, Thymine (T) imino nitrogen (N3): pKa $\approx$ 9.7), which means such DNA protonation or deprotonation processes as contributing factors to the observed separation outcomes are excluded.

c) Inconsistent modulation trend for anions:

In Fig. S4 the partition results were categorized into four distinct groups based on cation types to systematically evaluate anion modulation capabilities. This comparison reveals that anion-

mediated effects exhibit markedly lower consistency compared to cation-dominated regulation across the four groups. For instance, as shown in the PEG/sodium salt systems versus PEG/lithium counterparts, the relative ordering of citrate and sulfate anions are inverted (e.g., citrate > sulfate in Na^+ systems vs. sulfate > citrate in Li^+ systems). Similar inversions were also observed in other comparisons. However, for a fixed cation, the pH order of different salts is consistent (sulfate < tartrate < citrate). This contrast underscores the predominance of cation-specific interactions over pH-mediated effects in governing the separation outcomes.

d) The minimal pH variation in the current experimental design:

The optimization process shown in Fig. 2 focused on tuning K^+/Na^+ ratios while maintaining small variations in pH (e.g., pH=6.63 for sodium phosphate buffer and H=6.80 for potassium phosphate buffer), successfully achieving the separation of single-chirality (6,5) and (8,3).

18. Donnan potential measurement

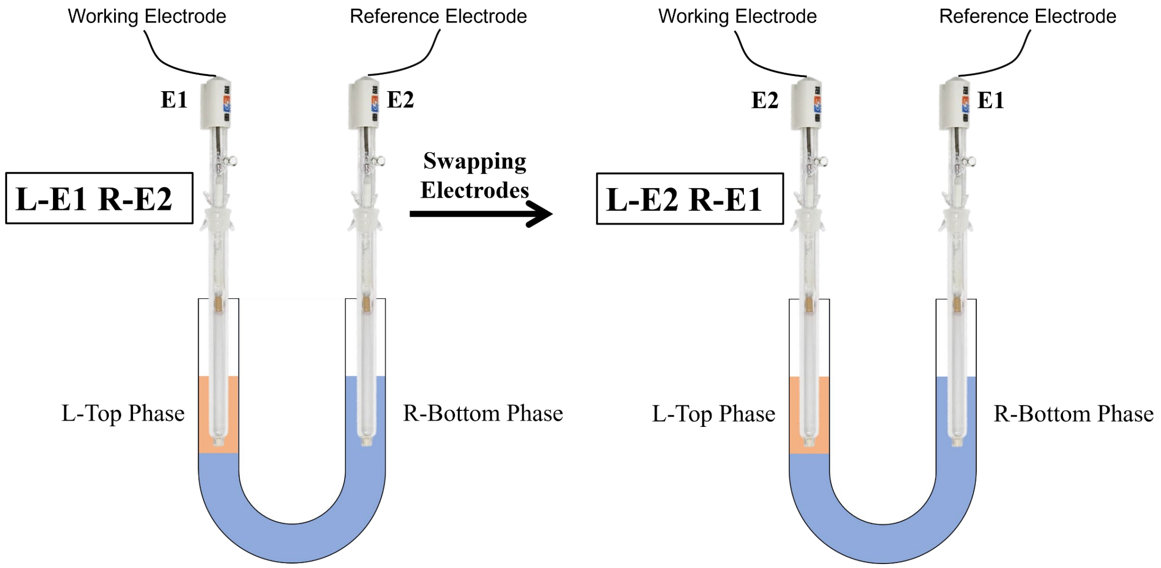

Fig. S28. Schemes illustrating the electrode settings for the measurement of Donnan potential at the interface of PEG/salt aqueous two-phase systems.

Below from Fig. S29 to Fig. S35 are the electric potential data obtained during the Donnan potential measurement of seven sets of PEG/salt ATP systems.

1) Donnan potential of PEG/potassium phosphate ATP system

Left panel (Fig. S29A) is the original electric potential data obtained during the Donnan potential measurement of the PEG/potassium phosphate ATP system. The right panel, as depicted in Fig. S29B, presents the short-circuit potential data encompassing the last 600 seconds, while also including the Donnan potential measurement data for the last 1000 seconds of the PEG/potassium phosphate ATP system once stability is achieved, both of which are derived from the data set illustrated in the left panel.

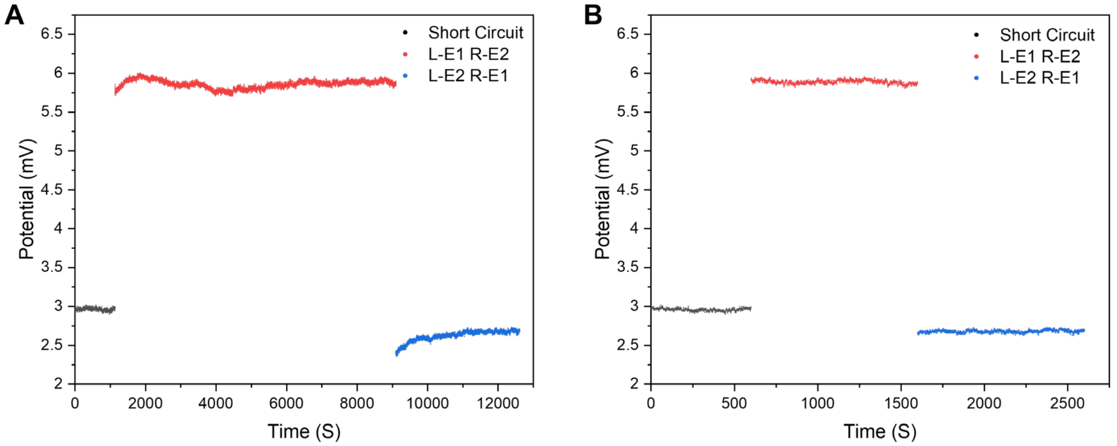

Fig. S29. Donnan potential measurement of PEG/potassium phosphate ATP system. (**A**) Original electric potential data. (**B**) Data after reaching stability (last 600 seconds for short circuit and last 1000 seconds for the measurement of PEG/potassium phosphate ATP system) extracted from part A.

2) Donnan potential of PEG/sodium phosphate ATP system

Left panel (Fig. S30A) is the original electric potential data obtained during the Donnan potential measurement of the PEG/sodium phosphate ATP system. The right panel, as depicted in Fig. S30B, presents the short-circuit potential data encompassing the last 600 seconds, while also including the Donnan potential measurement data for the last 1000 seconds of the PEG/sodium phosphate ATP system once stability is achieved, both of which are derived from the data set illustrated in the left panel.

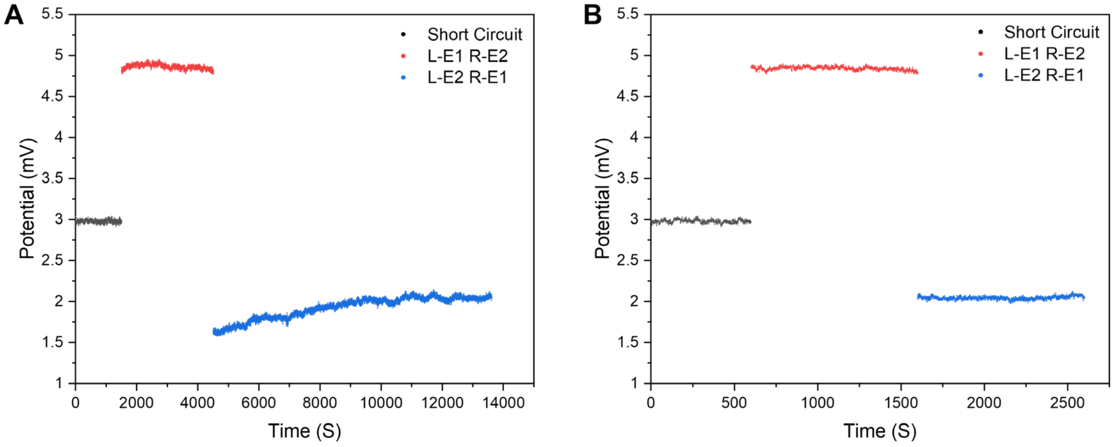

Fig. S30. Donnan potential measurement of PEG/sodium phosphate ATP system. (**A**) Original electric potential data. (**B**) Data after reaching stability (last 600 seconds for short circuit and last 1000 seconds for the measurement of PEG/sodium phosphate ATP system) extracted from part A.

3) Donnan potential of PEG/ammonium phosphate ATP system

Left panel (Fig. S31A) is the original electric potential data obtained during the Donnan potential measurement of the PEG/ammonium phosphate ATP system. The right panel, as depicted in Fig. S31B, presents the short-circuit potential data encompassing the last 600 seconds, while also including the Donnan potential measurement data for the last 1000 seconds of the PEG/ammonium

phosphate ATP system once stability is achieved, both of which are derived from the data set illustrated in the left panel.

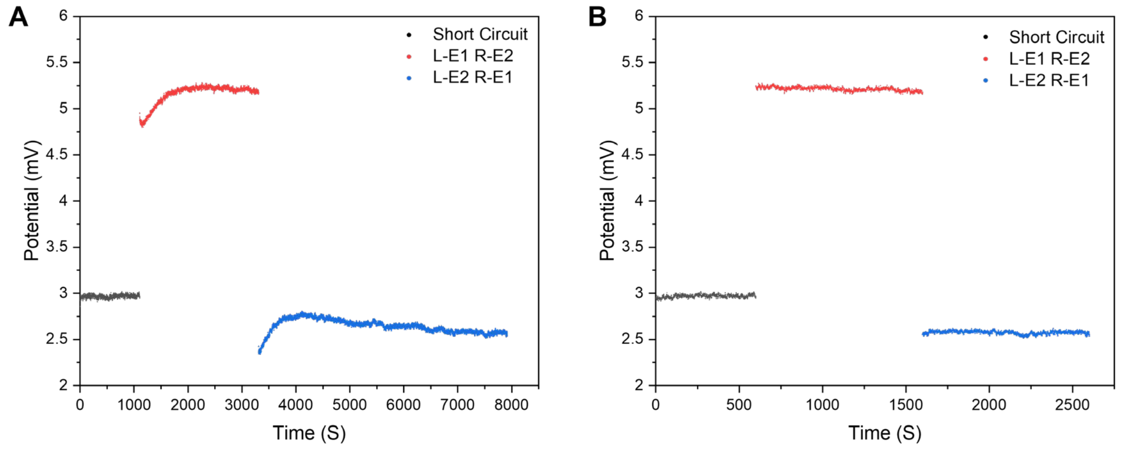

Fig. S31. Donnan potential measurement of PEG/ammonium phosphate ATP system. (**A**) Original electric potential data. (**B**) Data after reaching stability (last 600 seconds for short circuit and last 1000 seconds for the measurement of PEG/ammonium phosphate ATP system) extracted from part A.

4) Donnan potential of PEG/potassium citrate ATP system

Left panel (Fig. S32A) is the original electric potential data obtained during the Donnan potential measurement of the PEG/potassium citrate ATP system. The right panel, as depicted in Fig. S32B, presents the short-circuit potential data encompassing the last 600 seconds, while also including the Donnan potential measurement data for the last 1000 seconds of the PEG/potassium citrate ATP system once stability is achieved, both of which are derived from the data set illustrated in the left panel.

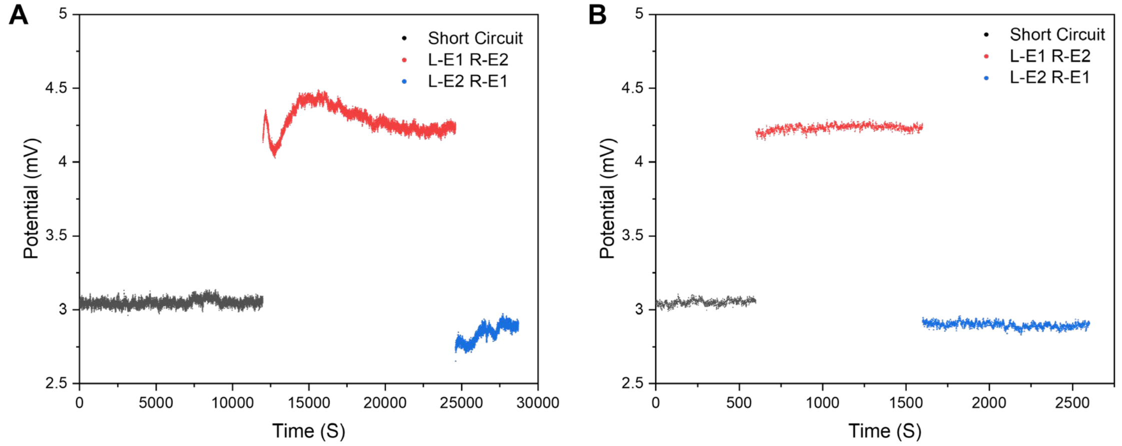

Fig. S32. Donnan potential measurement of PEG/potassium citrate ATP system. (**A**) Original electric potential data. (**B**) Data after reaching stability (last 600 seconds for short circuit and last 1000 seconds for the measurement of PEG/potassium citrate ATP system) extracted from part A.

5) Donnan potential of PEG/sodium citrate ATP system

Left panel (Fig. S33A) is the original electric potential data obtained during the Donnan potential measurement of the PEG/sodium citrate ATP system. The right panel, as depicted in Fig. S33B,

presents the short-circuit potential data encompassing the last 600 seconds, while also including the Donnan potential measurement data for the last 1000 seconds of the PEG/sodium citrate ATP system once stability is achieved, both of which are derived from the data set illustrated in the left panel.

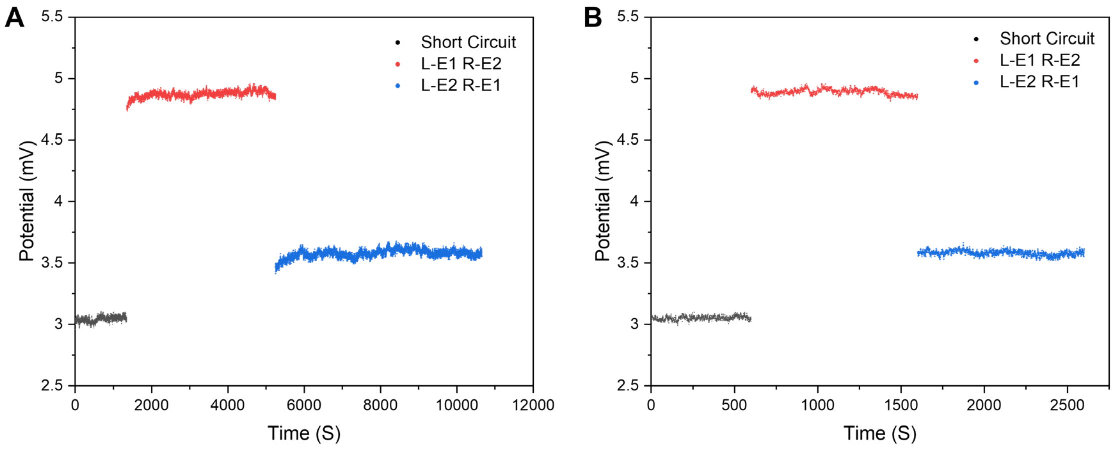

Fig. S33. Donnan potential measurement of PEG/sodium citrate ATP system. (**A**) Original electric potential data. (**B**) Data after reaching stability (last 600 seconds for short circuit and last 1000 seconds for the measurement of PEG/sodium citrate ATP system) extracted from part A.

6) Donnan potential of PEG/lithium citrate ATP system

Left panel (Fig. S34A) is the original electric potential data obtained during the Donnan potential measurement of the PEG/lithium citrate ATP system. The right panel, as depicted in Fig. S34B, presents the short-circuit potential data encompassing the last 600 seconds, while also including the Donnan potential measurement data for the last 1000 seconds of the PEG/lithium citrate ATP system once stability is achieved, both of which are derived from the data set illustrated in the left panel.

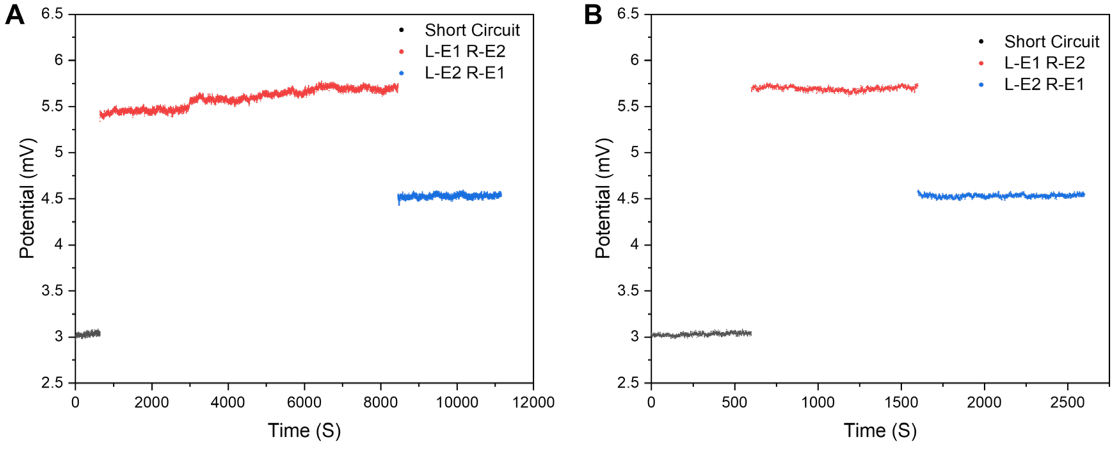

Fig. S34. Donnan potential measurement of PEG/lithium citrate ATP system. (**A**) Original electric potential data. (**B**) Data after reaching stability (last 600 seconds for short circuit and last 1000 seconds for the measurement of PEG/lithium citrate ATP system) extracted from part A.

7) Donnan potential of PEG/ammonium citrate ATP system

Left panel (Fig. S35A) is the original electric potential data obtained during the Donnan potential measurement of the PEG/ammonium citrate ATP system. The right panel, as depicted in Fig. S35B, presents the short-circuit potential data encompassing the last 600 seconds, while also including the Donnan potential measurement data for the last 1000 seconds of the PEG/ammonium citrate ATP system once stability is achieved, both of which are derived from the data set illustrated in the left panel.

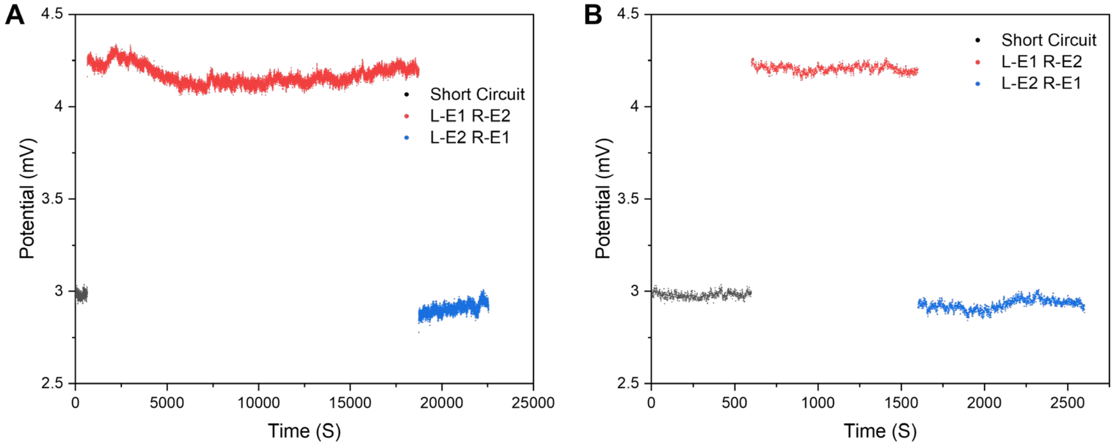

Fig. S35. Donnan potential measurement of PEG/ammonium citrate ATP system. (**A**) Original electric potential data. (**B**) Data after reaching stability (last 600 seconds for short circuit and last 1000 seconds for the measurement of PEG/ammonium citrate ATP system) extracted from part A.
